# Annotated checklist of the amphibians and reptiles of Zacatecas, Mexico

**DOI:** 10.3897/zookeys.1281.174112

**Published:** 2026-06-02

**Authors:** José Jesús Sigala-Rodríguez, Rubén Alonso Carbajal-Márquez, Alondra Encarnación-Luévano, Jesús Lenin Lara-Galván, Jorge Alberto Bañuelos-Alamillo, Eric Centenero-Alcalá, Iván Trinidad Ahumada-Carrillo, Fahd Henrry Carmona-Torres

**Affiliations:** 1 Colección Zoológica, Departamento de Biología, Universidad Autónoma de Aguascalientes, Av. Universidad 940, Ciudad Universitaria 20131, Aguascalientes, Mexico Laboratorio de Sistemática Molecular, Carrera de Biología, UMIEZ, Facultad de Estudios Superiores Zaragoza, Universidad Nacional Autónoma de México Ciudad de México Mexico; 2 Laboratorio de Biología, Centro de Bachillerato Tecnológico Agropecuario No. 167 "Gral. J. Jesús González Ortega", Km 1 Camino a Agua Fría, Col. Agua Fría 99253, Valparaíso, Zacatecas, Mexico Departamento de Etología, Fauna Silvestre y Animales de Laboratorio, Facultad de Medicina Veterinaria y Zootecnia, Universidad Nacional Autónoma de México Ciudad de México Mexico; 3 Laboratorio de Sistemática Molecular, Carrera de Biología, UMIEZ, Facultad de Estudios Superiores Zaragoza, Universidad Nacional Autónoma de México, Calle Batalla 5 de Mayo s/n, Ejército de Oriente 09230, Ciudad de México, Mexico Colección Zoológica, Departamento de Biología, Universidad Autónoma de Aguascalientes Aguascalientes Mexico; 4 Herp.Mx A.C., 28989, Villa Álvarez, Colima, Mexico Laboratorio de Biología, Centro de Bachillerato Tecnológico Agropecuario No. 167 "Gral. J. Jesús González Ortega" Zacatecas Mexico; 5 Biodiversa A. C., Avenida de la Rivera 203,45900, Chapala, Jalisco, Mexico Biodiversa A. C. Jalisco Mexico; 6 Departamento de Etología, Fauna Silvestre y Animales de Laboratorio, Facultad de Medicina Veterinaria y Zootecnia, Universidad Nacional Autónoma de México, Av. Universidad 3000, Ciudad Universitaria 04510, Coyoacán, Ciudad de México, Mexico Herp.Mx A.C. Villa Álvarez Mexico

**Keywords:** Amphibians, biodiversity, checklist, herpetofauna, Mexico, reptiles, Zacatecas

## Abstract

Zacatecas is in the north-central region of Mexico. Its territorial extension, as well as the unique combination of climatic and physiographic characteristics, favor high biological diversity. However, until now, it has been considered one of the states with lowest herpetofaunal diversity, mainly due to low sampling effort. We provide an updated checklist of the herpetofauna of Zacatecas, including the physiographic provinces and ecoregions where they occur, a summary of their conservation status, and a comparison with neighboring states. Zacatecas has 25 species of native amphibians and 119 native reptiles, with five introduced species (1 frog, 2 lizards, 1 snake and 1 turtle). Of 149 species, four have their type locality in Zacatecas, 16 represent new state records, and 232 records from 79 species are new municipal contributions. More than half of the native herpetofauna (53.5%) of Zacatecas is endemic to Mexico. However, it does not have state endemics, but harbors populations of regional endemics that only inhabit north-central Mexico. Of the native amphibian and reptile species in Zacatecas, 2.8% are listed by IUCN in threatened categories (i.e., Vulnerable, Endangered, Critically Endangered), 13.9% are placed in a protected category by SEMARNAT (i.e., Threatened and in Danger of Extinction), and 33.3% are categorized as high vulnerability by the EVS criteria. Among adjacent states with which it shares a border, Zacatecas is the fourth with the largest territorial extension, the fifth in herpetofauna diversity, and the third with the highest number of country endemic species. Large areas of the state remain underexplored, suggesting that the herpetofauna richness of Zacatecas may increase in the future.

## Introduction

The state of Zacatecas is considered one of the least studied states and with the lowest richness of amphibians and reptiles in the country (Flores-Villela and Pérez-Mendoza 2006; Ochoa-Ochoa and Flores-Villela 2006). However, the study of herpetofauna in Zacatecas dates back at least 157 years, with records of species such as *Crotalus atrox* by Dugès (1869), *Sceloporus minor* (Cope 1885), *Hypsiglena affinis* (Boulenger 1894) and *Craugastor occidentalis* (Boulenger 1898). Between the years 1900–1950, the records of species made for the state in the works of Smith (1938a, 1938b, 1939) and Smith and Taylor (1945, 1948, 1950) stand out. The most notable contributions, between the years 1950–2000, are those made by Chrapliwy (1956), Baker et al. (1967, 1981), Wilson and McCranie (1979), Webb (1980, 1982), and Bezy and Flores-Villela (1999). Finally, from the 2000s onwards, the registration of new species for Zacatecas continued, highlighting the contributions made by Ahumada-Carrillo et al. (2011, 2014), and Bañuelos-Alamillo et al. (2015, 2016, 2017). In a study on the biodiversity of amphibians and reptiles from Mexico, Parra-Olea et al. (2014) mention 14 species of amphibians and Flores-Villela and García-Vázquez (2014) reported 64 reptiles for Zacatecas, giving a total of 78 species. Recently, the number of herpetofauna species in Zacatecas was updated, with Sigala-Rodríguez et al. (2020a, 2020b) reporting 25 species of amphibians and 108 reptiles. Nevertheless, Ramírez-Bautista et al. (2023) report a richness of only 118 species of herpetofauna. Therefore, an updated and verified checklist is needed for Zacatecas.

In this work, we present in detail the formal checklist of amphibians and reptiles from Zacatecas, after conducting an exhaustive search of historical information (specialized literature), review of museum specimens, electronic databases, and fieldwork in the last three decades. The herpetofauna of Zacatecas is characterized based on ecosystem affinities and conservation status. Likewise, a comparison is made with the states adjacent to Zacatecas.

## Materials and methods

### Study area

**Figure 1.**
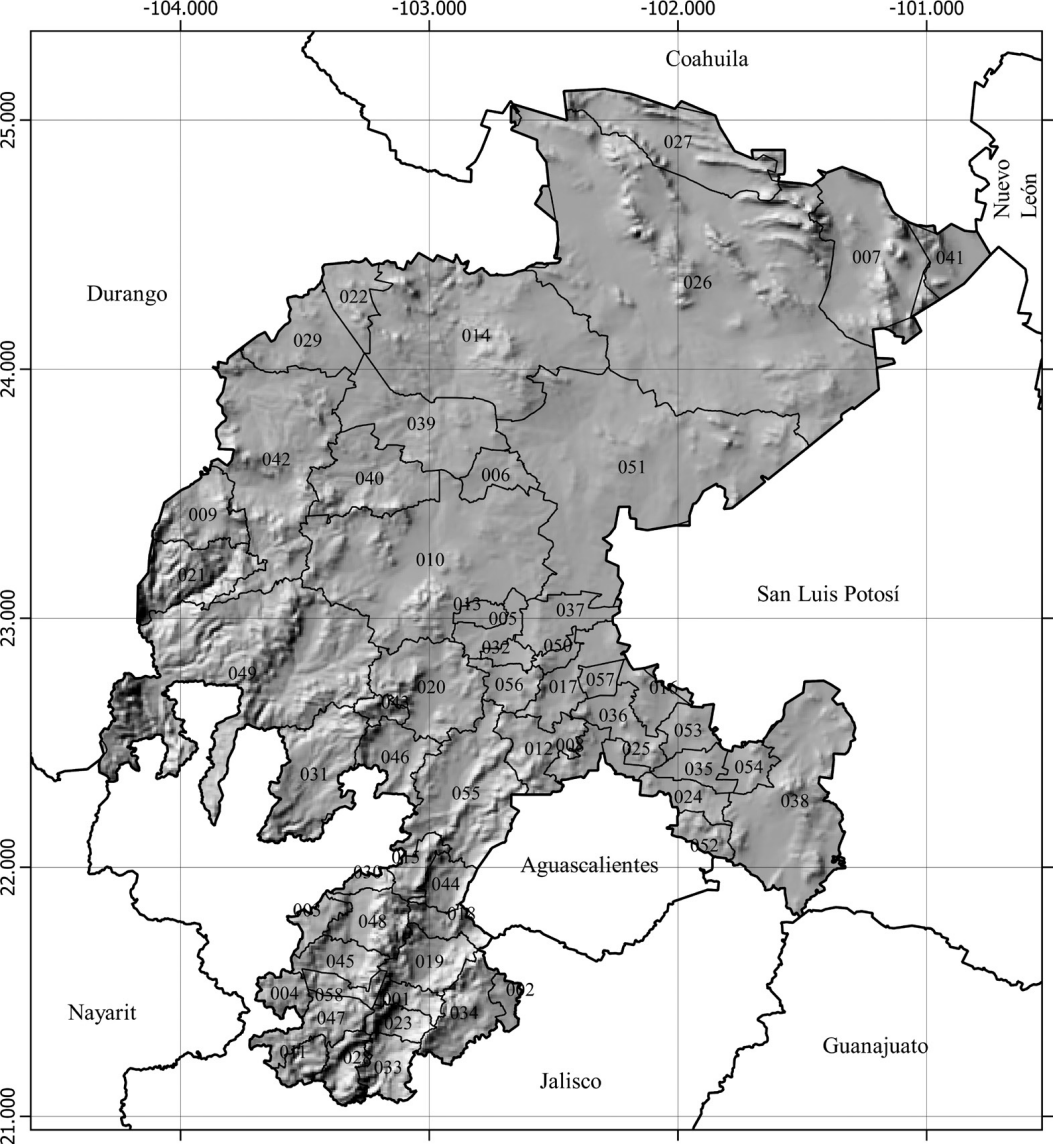
Geographical description of the state of Zacatecas in Mexico. The relief of the state is colored with gray shading and was based on the digital elevation model GMTED2010 (DOI/USGS/EROS 2010). Located at the north-central region of the country, Zacatecas borders the states of Aguascalientes, Coahuila, Durango, Guanajuato, Jalisco, Nayarit, Nuevo León, and San Luis Potosí. In order to identify the new municipalities and state contributions of herpetofauna we present the municipality division (INEGI 2022). Abbreviations: 001 = Apozol, 002 = Apulco, 003 = Atolinga, 004 = Benito Juárez, 005 = Calera, 006 = Cañitas de Felipe Pescador, 007 = Concepción del Oro, 008 = Cuauhtémoc, 009 = Chalchihuites, 010 = Fresnillo, 011 = Trinidad García de la Cadena, 012 = Genaro Codina, 013 = General Enrique Estrada, 014 = General Francisco R. Murguía, 015 = El Plateado de Joaquín Amaro, 016 = General Pánfilo Natera, 017 = Guadalupe, 018 = Huanusco, 019 = Jalpa, 020 = Jerez, 021 = Jiménez del Teúl, 022 = Juan Aldama, 023 = Juchipila, 024 = Loreto, 025 = Luis Moya, 026 = Mazapil, 027 = Melchor Ocampo, 028 = Mezquital del Oro, 029 = Miguel Auza, 030 = Momax, 031 = Monte Escobedo, 032 = Morelos, 033 = Moyahua de Estrada, 034 = Nochistlán de Mejía, 035 = Noria de Ángeles, 036 = Ojocaliente, 037 = Pánuco, 038 = Pinos, 039 = Río Grande, 040 = Sain Alto, 041 = El Salvador, 042 = Sombrerete, 043 = Susticacán, 044 = Tabasco, 045 = Tepechitlán, 046 = Tepetongo, 047 = Teúl de González Ortega, 048 = Tlaltenango de Sánchez Román, 049 = Valparaíso, 050 = Vetagrande, 051 = Villa de Cos, 052 = Villa García, 053 = Villa González Ortega, 054 = Villa Hidalgo, 055 = Villanueva, 056 = Zacatecas, 057 = Trancoso, 058 = Santa María de la Paz.

**Figure 2.**
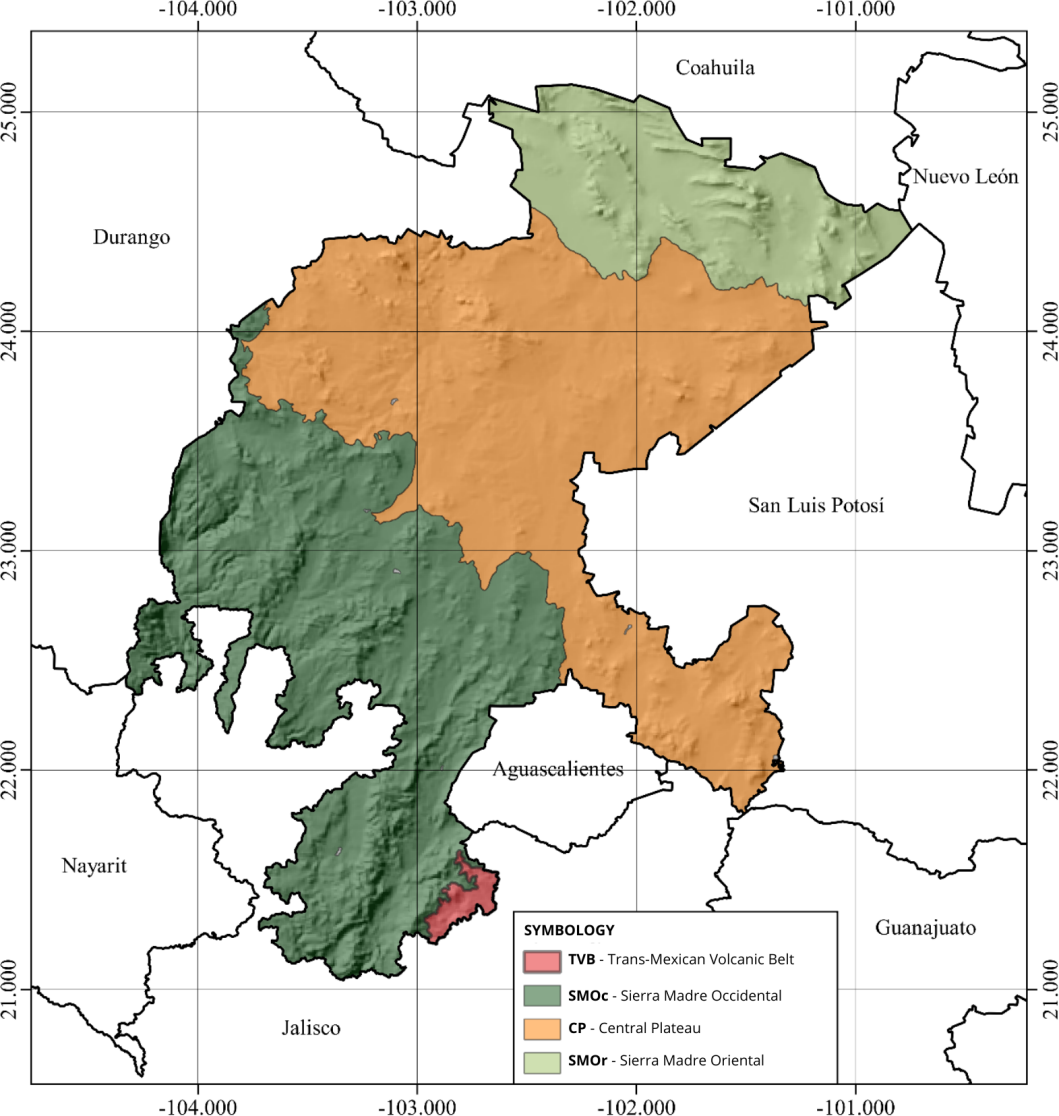
Physiographic provinces of Zacatecas state, Mexico, based on Cervantes-Zamora et al. (1990). The map shows the four provinces in the state. Abbreviations: TVB = Trans-Mexican Volcanic Belt, SMOc = Sierra Madre Occidental, CP = Central Plateau, SMOr = Sierra Madre Oriental.

With a territorial area of 75,275.3 km^2^, Zacatecas represents 3.8% of the surface of Mexico, placing it in tenth place nationally, regarding its area (INEGI 2021). It is located in Mexico's central northern region, with altitude ranging from 800 to 3,120 m a.s.l. and being divided into 58 municipalities (INEGI 2022) (Fig. 1). To the north it borders with Coahuila, to the northeast with Nuevo León, to the east with San Luis Potosí, to the southeast with Guanajuato, to the south with the states of Aguascalientes and Jalisco, to the northwest with Nayarit, and to the west with Durango (INEGI 2022) (Fig. 1). The state is located in important areas of biogeographic transitions, which includes the plains of the Central Plateau (CP) with characteristics typical of the great deserts of North America, to the north elevations remaining in the Sierra Madre Oriental (SMOr), to the west the mountain ranges of the great Sierra Madre Occidental Mountain range (SMOc) and, to the south, a small portion associated with the Trans-Mexican Volcanic Belt (TVB) (Fig. 2) (Cervantes-Zamora et al. 1990). According to the extension covered by the different types of soils, Leptosol, Phaeozem, and Calcisol stand out, which together represent 67.4% of the state territory (De la Trinidad-Cabrera 2020). The predominant climate in Zacatecas is dry (INEGI 2011); the average annual temperature is 16 °C with an average annual precipitation of 510 mm, with extreme variations in temperature greater than 30 °C (INEGI 2011). Seventy-five percent of its surface is considered arid with xeric vegetation and grasslands. Temperate climates prevail in the western part, mainly associated with subtropical scrub, chaparral, and grasslands, and, to a lesser extent, with temperate forest located in mountainous areas. Semi-warm and warm climates are restricted to small fragments in the extreme south and southwest, giving rise to tropical forests and grasslands. Temperate forests cover a good portion of the territory to the west in the SMOc and TVB, but also to the northeast in the low mountain ranges of the SMOr and the isolated elevations of the CP. Finally, branches of dry Pacific forests enter the SMOc canyons (De la Trinidad-Cabrera 2020).

### Data collection

We compiled a list of amphibians and reptiles found in Zacatecas through a combination of our fieldwork during the last three decades, a thorough literature review, and examination of historical records in biological collections. The amphibian scientific names were established using taxonomic data from Frost (2025), while the reptile scientific names were established using taxonomic data from O’Connell and Smith (2018), Leaché et al. (2021) and Uetz et al. (2025). The individuals were either photographed or collected and then deposited in the specimens and photographic collections at the Colección Zoológica of the Universidad Autónoma de Aguascalientes (CZUAA), Colección Nacional de Anfibios y Reptiles (CNAR) of the Universidad Nacional Autónoma de México, University of Texas at Arlington Digital Collection (UTADC), and San Diego Natural History Museum (SDNHM). The permits for the collection of specimens were issued to José Jesús Sigala-Rodríguez and are as follows: Licencia de Colector Científico FAUT-110, DOO 02.-3614 99, NUM/SGPA/DGVS/04325 04, NUM/SGPA/DGVS/06292/06, SGPA/DGVS/05874/17, SGPA/DGVS/7494/19, and SGPA/DGVS/07154/21. We retrieved records for all the species from biological collections through online national and international repositories, including The Global Biodiversity Information Facility (GBIF 2025), iNaturalistMX (iNaturalist 2025), Sistema Nacional de Información sobre Biodiversidad de México (SNIB) (CONABIO 2021), and Distributed Databases with Backbone, in this case, Vertebrate Networks (VertNet 2025). The list of the biological collections from which we retrieved data is available in Suppl. material 1. The data for each species was cleaned using biological, distributional, ecological, life history, and taxonomic data. To display the geographic correlation of each data, we employed QGIS 2.18.14 (QGIS 2009) and ArcGIS 10.5.1 (ESRI 2016) Geographic Information System (GIS).

**Figure 3.**
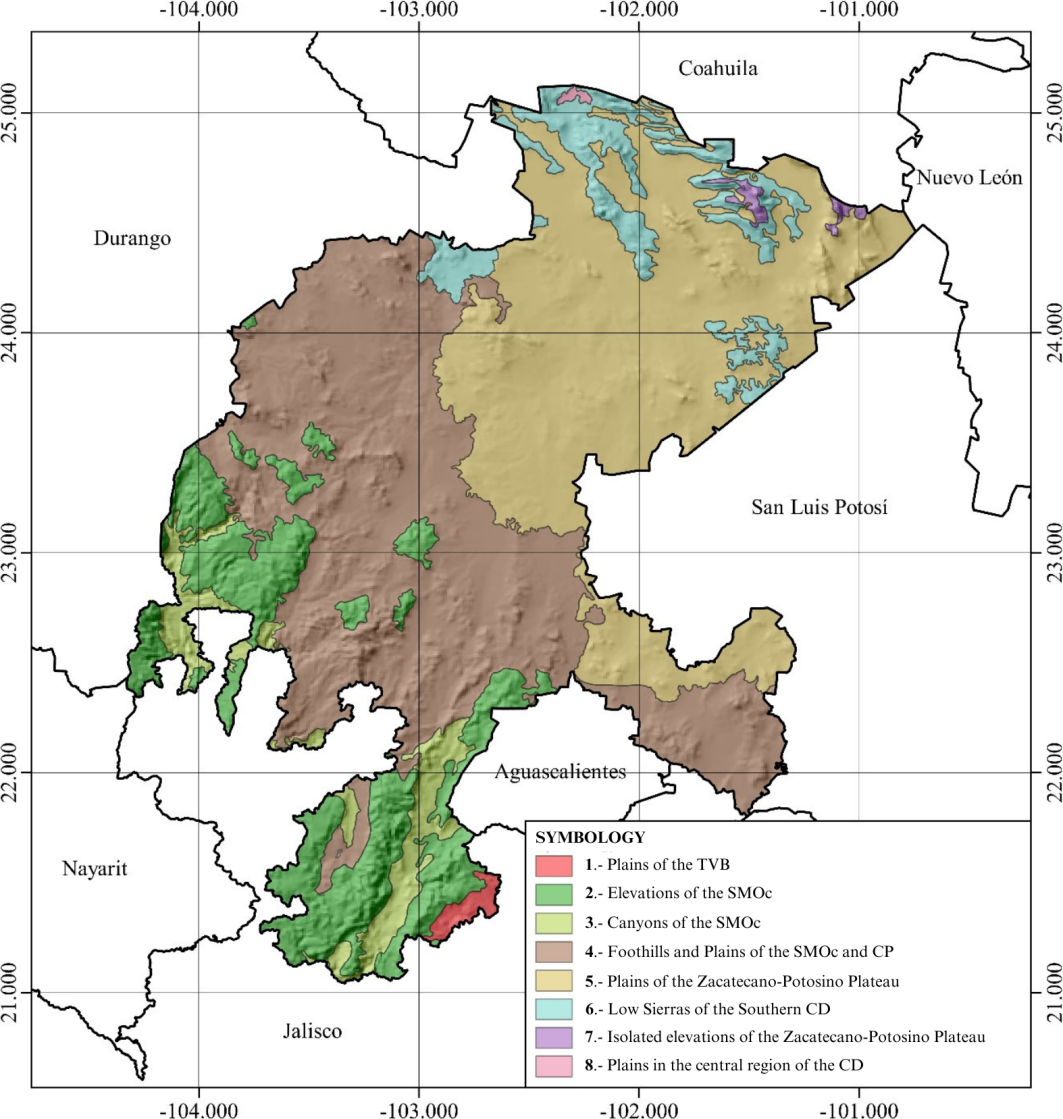
Ecoregions of Zacatecas state, Mexico, based on INEGI et al. (2008). The map shows the eight ecoregions in the state: 1. Plains of the Trans-Mexican Volcanic Belt, 2. Elevations of the Sierra Madre Occidental, 3. Canyons of the Sierra Madre Occidental, 4. Foothills and Plains of the Sierra Madre Occidental and the Central Plateau, 5. Plains of the Zacatecano-Potosino Plateau, 6. Low Sierras of the Southern Chihuahua Desert, 7. Isolated elevations of the Zacatecano-Potosino Plateau, and 8. Plains in the central region of the Chihuahuan Desert.

We present the revised herpetofauna list of Zacatecas which includes new state and municipal species contributions, its regionalization based on the geographical correspondence of the records with the physiographic provinces, SMOr (11,132.66 km^2^ = 14.78%), SMOc (29,080.29 km^2^ = 39.04%), TVB (625.74 km^2^ = 1.05%), CP (33,612.84 km^2^ = 45.13%) (Fig. 2) (Cervantes-Zamora et al. 1990; De la Trinidad-Cabrera 2020), and the ecoregions, 1. Plains of the Trans-Mexican Volcanic Belt, 2. Elevations of the Sierra Madre Occidental, 3. Canyons of the Sierra Madre Occidental, 4. Foothills and Plains of the Sierra Madre Occidental and the Central Plateau, 5. Plains of the Zacatecano-Potosino Plateau, 6. Low Sierras of the Southern Chihuahua Desert, 7. Isolated elevations of the Zacatecano-Potosino Plateau, and 8. Plains in the central regions of the Chihuahuan Desert (Fig. 3) (INEGI et al. 2008). We present the conservation status for the herpetofauna, considering the International Union for Conservation of Nature (IUCN 2025), the Environmental Vulnerability Score (EVS) (Wilson et al. 2013a, 2013b) and the conservation status in Mexico according to SEMARNAT (2010). We compiled a list of potential species based on records near the state line and in ecoregions that had continuity within the state. Finally, we present a comparison with adjacent states with which Zacatecas shares a border.

## Results

**Figure 4.**
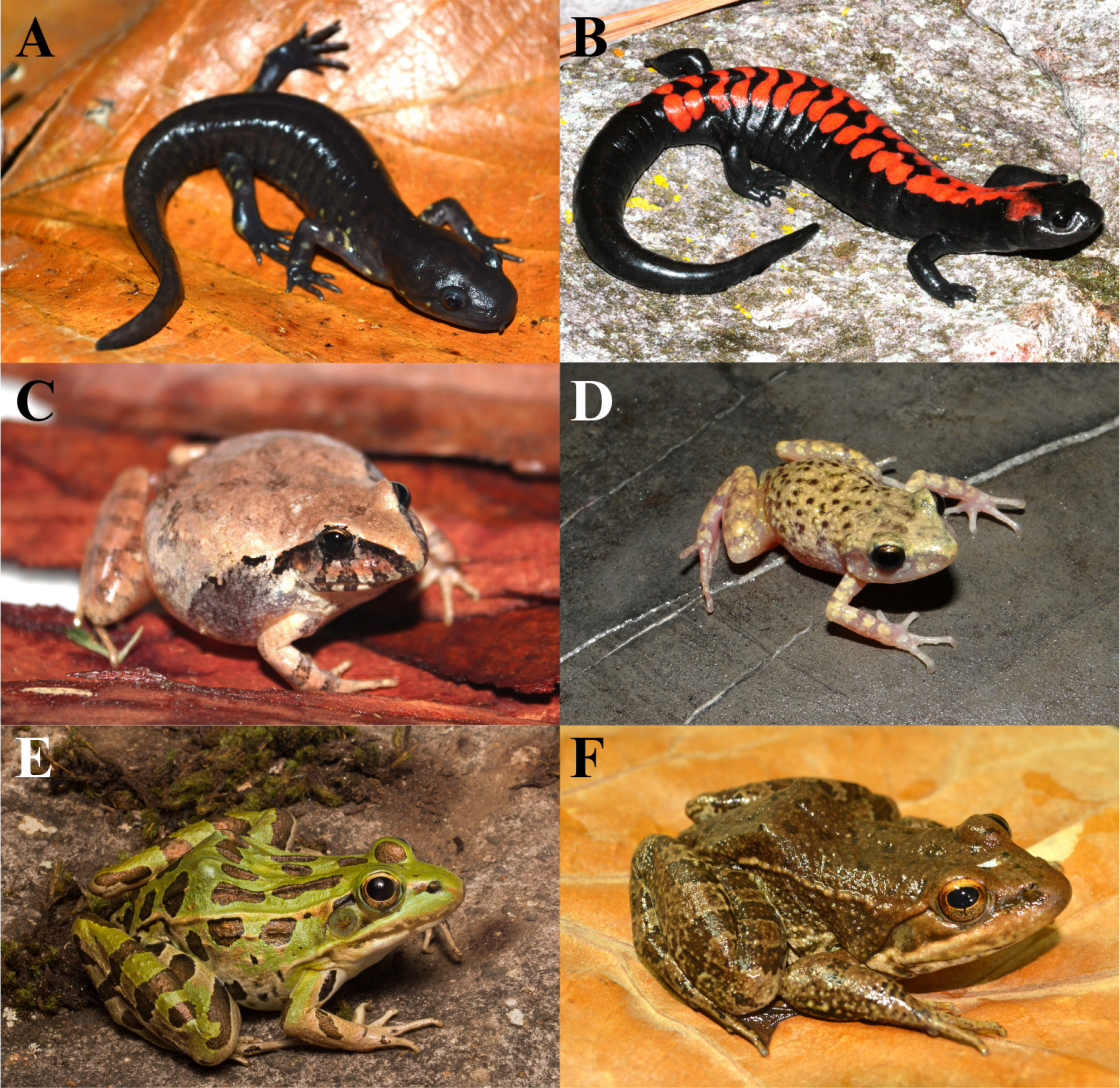
Representative amphibians present in Zacatecas, Mexico. **A***Ambystoma rosaceum*; **B**. *Isthmura bellii*; **C**. *Craugastor occidentalis*; **D**. *Eleutherodactylus guttilatus*; **E**. *Lithobates berlandieri*; **F**. *Lithobates psilonota*. Photos by Jorge A. Bañuelos-Alamillo (**A, C, F**), Iván T. Ahumada-Carrillo (**B, E**), and Chris Grünwald (**D**).

**Figure 5.**
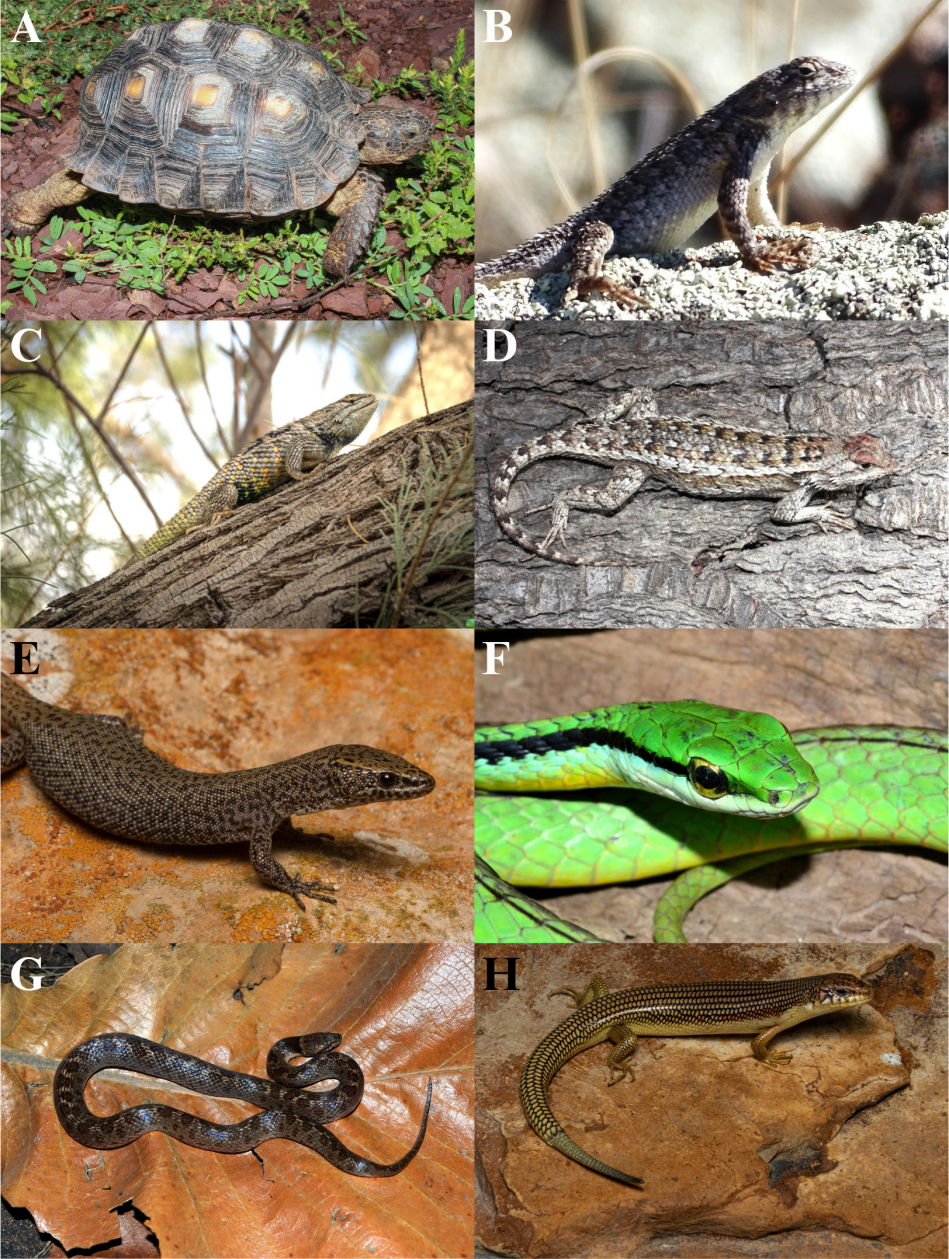
Representative reptiles present in Zacatecas, Mexico. **A***Gopherus berlandieri*; **B**. *Sceloporus albiventris*; **C**. *Sceloporus bimaculosus*; **D**. *Sceloporus olivaceus*; **E**. *Xantusia sanchezi*; **F**. *Leptophis diplotropis*; **G**. *Hypsiglena affinis*; **H**. *Plestiodon obsoletus*. Photos by Iván T. Ahumada-Carrillo (**A, B, E**), Eric Centenero-Alcalá (**C, D, H**), J. Jesús Sigala-Rodríguez (**F**) and Jorge A. Bañuelos-Alamillo (**G**).

Zacatecas is home to 149 amphibians and reptiles including five introduced species. In Table 1, we summarize the list of herpetofauna recorded by different sources (see Suppl. material 2) as well as those new contributions. Native amphibians and reptiles are represented by 144 species: 25 amphibians (three salamanders and 22 anurans) (Fig. 4), and 119 reptiles (four turtles, 51 lizards and 64 snakes) (Fig. 5). The introduced species are the Common Bullfrog (*Aquarana catesbeiana*), the Common House Gecko (*Hemidactylus frenatus*), the Mediterranean House Gecko (*H. turcicus*), the Red-eared Slider (*Trachemys scripta*), and the Brahminy Blindsnake (*Indotyphlops braminus*). The species recorded by our fieldwork include two amphibians and ten reptiles, those from collection records are four, two lizards and two snakes (*Hemidactylus turcicus*, *Sceloporus bimaculosus*, *Sonora semiannulata* and *Rena dugesii*) resulting in 16 new state contributions; and those previously recorded in the literature include 24 amphibians and 109 reptiles.

**Table 1.** Amphibians and reptiles of the state of Zacatecas with distributional and conservation status. Abbreviations: GD = Global Distribution; E = Endemic to Mexico; NE = No endemic to Mexico; IN = Introduced. PP = Physiographic Province: TVB = Trans-Mexican Volcanic Belt; SMOc = Sierra Madre Occidental; CP = Central Plateau; SMOr = Sierra Madre Oriental. ECOR = Ecoregions: 1 = Plains of the Trans-Mexican Volcanic Belt; 2 = Elevations of the Sierra Madre Occidental; 3 = Canyons of the Sierra Madre Occidental; 4 = Foothills and Plains of the Sierra Madre Occidental and the Central Plateau; 5 = Plains of the Zacatecano-Potosino Plateau; 6 = Low Sierras of the Southern Chihuahua Desert; 7 = Isolated elevations of the Zacatecano-Potosino Plateau; 8 = Plains in the central region of the Chihuahuan Desert. IUCN Status: DD = Data Deficient; LC = Least Concern; VU = Vulnerable; NT = Near Threatened; EN = Endangered; CR = Critically Endangered; NE = Not Evaluated (IUCN 2025). EVS = Environmental Vulnerability Score (the higher the score the greater the vulnerability): NL = Not Listed; Low (L) vulnerability species (EVS of 3–9); medium (M) vulnerability species (EVS of 10–13); high (H) vulnerability species (EVS of 14–20) (Wilson et al. 2013a, 2013b). Conservation status in Mexico according to SEMARNAT (2010): NL = Not Listed; Pr = Subject to Special Protection; P = In Danger of Extinction; A = Threatened. SOURCE: reference to the first record in the state, Suppl. material 2 completes the information for each reference.

Taxa	GD	PP	ECOR	IUCN	EVS	SEMARNAT	Source
CLASS AMPHIBIA							
ORDER CAUDATA							
FAMILY AMBYSTOMATIDAE							
* Ambystoma rosaceum* Taylor, 1941	E	SMOc	2, 4	LC	14	Pr	Anderson and Webb (1978)
* Ambystoma velasci* (Dugès, 1888)	E	SMOc, CP	2, 4, 6	LC	10	Pr	Dundee and Liner (1976)
FAMILY PLETHODONTIDAE							
* Isthmura bellii* (Gray, 1850)	E	SMOc	2	LC	12	A	Wilson and McCranie (1979)
ORDER ANURA							
FAMILY BUFONIDAE							
* Anaxyrus cognatus* (Say, 1822)	NE	CP, SMOr	4,5, 6	LC	8	NL	Krupa (1990)
* Anaxyrus compactilis* (Wiegmann, 1833)	E	TVB, SMOc	1, 2, 4	LC	14	NL	Smith and Taylor (1948)
* Anaxyrus debilis* (Girard, 1854)	NE	CP, SMOr	4, 5	LC	7	Pr	Taylor (1936)
* Anaxyrus punctatus* (Baird & Girard, 1852)	NE	SMOc, CP, SMOr	1, 2, 3, 4, 5, 6, 7	LC	5	NL	Chrapliwy (1956)
* Incilius occidentalis* (Camerano, 1879)	E	SMOc, CP	2, 3, 4	LC	11	NL	Kellogg (1932)
* Rhinella horribilis* (Wiegmann, 1833)	NE	SMOc	3	LC	3	NL	Ahumada-Carrillo et al. (2011)
FAMILY CRAUGASTORIDAE							
* Craugastor augusti* (Dugès, 1879)	NE	SMOc, SMOr	2, 3, 6, 7	LC	8	NL	Smith and Taylor (1948)
* Craugastor occidentalis* (Taylor, 1941)	E	SMOc	2	LC	13	NL	Boulenger (1898)
FAMILY ELEUTHERODACTYLIDAE							
* Eleutherodactylus jamesdixoni* Devitt, Tseng, Taylor-Adair, Koganti, Timugura & Cannatella, 2023	E	SMOc	2, 4	NE	NL	NL	Grünwald et al. (2021)
* Eleutherodactylus guttilatus* (Cope, 1879)	NE	CP	5	LC	11	NL	This study
FAMILY HYLIDAE							
* Agalychnis dacnicolor* (Cope, 1864)	E	SMOc	3	LC	13	NL	Villalobos-Juárez & García-Padilla (2023)
* Dryophytes arenicolor* (Cope, 1866)	NE	TVB, SMOc, CP	2, 3, 4	LC	7	NL	Kellogg (1932)
* Dryophytes eximius* (Baird, 1854)	E	TVB, SMOc	2, 3, 4	LC	10	NL	Smith and Taylor (1948)
* Smilisca fodiens* (Boulenget, 1882)	NE	SMOc	2	LC	8	NL	Ahumada-Carrillo et al. (2011)
* Smilisca dentata* (Smith, 1957)	E	TVB	1	EN	14	A	Villalobos-Juárez (2023)
FAMILY MICROHYLIDAE							
* Hypopachus variolosus* (Cope, 1866)	NE	TVB, SMOc	2, 3, 4	LC	4	NL	Ahumada-Carrillo et al. (2011)
FAMILY RANIDAE							
* Aquarana catesbeiana* (Shaw, 1802)	IN	TVB, SMOc	1, 3	IN	IN	IN	This study
* Lithobates berlandieri* (Baird, 1859)	NE	SMOc, CP	2, 4	LC	7	Pr	Ahumada-Carrillo et al. (2011)
* Lithobates magnaocularis* (Frost & Bagnara, 1974)	E	SMOc	2, 3	LC	12	NL	Quintero-Díaz et al. (2015)
* Lithobates montezumae* (Baird, 1854)	E	TVB, SMOc, CP, SMOr	1, 2, 3, 4	LC	13	Pr	Kellogg (1932)
* Lithobates psilonota* (Webb, 2001)	E	SMOc	3	LC	14	NL	Ahumada-Carrillo et al. (2011)
FAMILY SCAPHIOPODIDAE							
* Scaphiopus couchii* Baird, 1854	NE	SMOc, CP, SMOr	4, 5	LC	3	NL	Smith and Taylor (1948)
* Spea multiplicata* (Cope, 1863)	NE	TVB, SMOc, CP, SMOr	1, 2, 3, 4, 5, 6	LC	6	NL	Smith and Taylor (1948)
CLASS REPTILIA							
ORDER TESTUDINES							
FAMILY EMYDIDAE							
* Terrapene nelsoni* Stejneger, 1925	E	SMOc	3	DD	18	Pr	Rosales-Martínez et al. (2022)
* Trachemys scripta* (Thunberg, 1792)	IN	SMOc	4	IN	IN	IN	Bañuelos-Alamillo and Osegueda-Berrios (2020)
FAMILY KINOSTERNIDAE							
* Kinosternon hirtipes* (Wagler, 1830)	NE	SMOc	4	LC	10	Pr	Iverson (1985)
* Kinosternon integrum* Le Conte, 1854	E	SMOc, CP, SMOr	1, 2, 3, 4, 5, 6	LC	11	Pr	Iverson (1998)
FAMILY TESTUDINAE							
* Gopherus berlandieri* Agassiz, 1857	NE	SMOr	5, 6	LC	18	A	This study
ORDER SQUAMATA							
SUBORDER LACERTILIA							
FAMILY ANGUIDAE							
* Barisia ciliaris* (Smith, 1942)	E	SMOc	2, 4	NE	15	NL	Smith (1942)
* Elgaria kingii* Gray, 1838	NE	SMOc	2	LC	10	Pr	Webb (1970)
* Gerrhonotus infernalis* Baird, 1859	NE	SMOc, SMOr	4, 5, 6	LC	13	NL	Campos-Rodríguez et al. (2004)
FAMILY ANOLIDAE							
* Anolis nebulosus* (Wiegmann, 1834)	E	SMOc, SMOr	2, 3, 4	LC	13	NL	Wilson and McCranie (1979)
FAMILY CROTAPHYTIDAE							
* Crotaphytus collaris* (Say, 1822)	NE	SMOr	5, 6	LC	13	A	Smith and Tanner (1974)
* Gambelia wislizenii* (Baird & Girard, 1852)	NE	SMOr	5, 6	LC	13	Pr	This study
FAMILY EUBLEPHARIDAE							
* Coleonyx brevis* Stejneger, 1893	NE	SMOr	6	LC	14	Pr	Baker et al. (1981)
FAMILY GEKKONIDAE							
* Hemidactylus frenatus* Duméril & Bribon, 1836	IN	SMOc	4	IN	IN	IN	Bañuelos-Alamillo et al. (2016)
* Hemidactylus turcicus* (Linnaeus, 1758)	IN	SMOc, CP, SMOr	4,5,6	IN	IN	IN	This study
FAMILY HELODERMATIDAE							
* Heloderma horridum* (Wiegmann, 1829)	E	SMOc	3	LC	11	A	Ávila-Villegas (2007)
FAMILY IGUANIDAE							
* Ctenosaura pectinata* (Wiegmann, 1834)	E	SMOc	3	LC	15	A	Baker et al. (1967)
FAMILY PHRYNOSOMATIDAE							
* Cophosaurus texanus* Troschel, 1852	NE	SMOr	5	LC	14	A	Morafka (1977)
* Holbrookia approximans* Baird, 1859	E	CP, SMOr	5, 6	NE	14	NL	Schmidt (1922)
* Phrynosoma cornutum* (Harlan, 1825)	NE	CP, SMOr	4, 5	LC	11	NL	Price (1990)
* Phrynosoma modestum* Girard, 1852	NE	CP, SMOr	2, 4, 5, 6	LC	12	NL	Smith (1935)
* Phrynosoma orbiculare* (Linnaeus, 1758)	E	SMOc, CP, SMOr	2, 4, 5, 6	LC	12	A	Smith and Taylor (1950)
* Phrynosoma ornatissimum* Girard, 1858	NE	SMOc	2, 4	NE	NL	NL	Flores-Villela et al. (1991)
* Sceloporus albiventris* Smith, 1939	E	SMOc	3	NE	16	NL	This study
* Sceloporus aurantius* Grummer & Bryson, 2014	E	SMOr	2, 4	NE	16	NL	Grummer and Bryson (2014)
* Sceloporus bimaculosus* Phelan & Brattstrom, 1955	NE	CP	5	NE	NL	NL	This study
* Sceloporus brownorum* Smith, Watkins-Colwell, Lemos-Espinal & Chiszar, 1997	E	SMOc, SMOr	2, 4	NE	14	NL	Smith (1939)
* Sceloporus cautus* Smith, 1938	E	CP	5	LC	15	NL	Smith (1938)
* Sceloporus clarkii* Baird & Girard, 1852	NE	SMOc	3	LC	10	NL	Baker et al. (1967)
* Sceloporus cowlesi* Lowe & Norris, 1956	NE	CP	5	NE	13	NL	Smith (1939)
* Sceloporus dugesii* Bocourt, 1874	E	SMOc	2, 4	LC	13	NL	Wilson and McCranie (1979)
* Sceloporus goldmani* Smith, 1937	E	CP	4	EN	15	NL	Carbajal-Márquez and Quintero-Díaz (2016)
* Sceloporus grammicus* Wiegmann, 1828	NE	SMOc, CP, SMOr	2, 4, 5, 6, 7	LC	9	Pr	Smith (1939)
* Sceloporus horridus* Wiegmann, 1834	E	SMOc	2, 3, 4	LC	11	NL	Smith (1939)
* Sceloporus huichol* Flores-Villela, Smith, Campillo-García, Martínez-Méndez & Campbell, 2022	E	SMOc	2, 3	NE	NL	NL	Campillo-García et al. (2023)
* Sceloporus jarrovii* Cope, 1875	NE	SMOc	2, 4	LC	11	NL	Smith (1938)
* Sceloporus maculosus* Smith, 1934	E	SMOr	6	VU	16	Pr	Baker et al. (1981)
* Sceloporus melanogaster* Cope, 1885	E	SMOc, CP, SMOr	2, 3, 4, 5, 6, 7	NE	NL	NL	Smith (1938)
* Sceloporus minor* Cope, 1885	E	CP, SMOr	4, 5, 6, 7	LC	14	NL	Cope (1885)
* Sceloporus olivaceus* Smith, 1934	NE	CP, SMOr	5	LC	13	NL	This study
* Sceloporus ornatus* Baird, 1859	E	SMOr	6	NT	16	NL	This study
* Sceloporus parvus* Smith, 1934	E	CP, SMOr	4, 5, 6, 7	LC	15	NL	Campos-Rodríguez et al. (2017)
* Sceloporus poinsetii* Baird & Girard, 1852	NE	SMOr	7	LC	12	NL	Chrapliwy (1956)
* Sceloporus * cf. *scalaris* Wiegmann, 1828	E	SMOr	5	LC	12	NL	This study
* Sceloporus shannonorum* Langebartel, 1959	E	SMOc	2	DD	15	NL	Ahumada-Carrillo et al. (2014)
* Sceloporus spinosus* Wiegmann, 1828	E	SMOc, CP, SMOr	4, 5	LC	12	NL	Smith (1939)
* Sceloporus utiformis* Cope, 1864	E	SMOc	3	LC	15	NL	Ahumada-Carrillo et al. (2011)
* Urosaurus bicarinatus* (Duméril, 1856)	E	SMOc	2, 3, 4	LC	12	NL	Baker et al. (1967)
* Uta stansburiana* Baird & Girard, 1852	NE	SMOr	6	LC	11	A	Ballinger and Tinkle (1972)
FAMILY PHYLLODACTYLIDAE							
* Phyllodactylus lanei* Smith, 1935	E	SMOc	3	LC	15	NL	Bañuelos-Alamillo et al. (2017a)
FAMILY SCINCIDAE							
* Plestiodon bilineatus* (Tanner, 1958)	E	SMOc	2	NE	13	NL	Feria-Ortíz et al. (2011)
* Plestiodon callicephalus* (Bocourt, 1879)	E	SMOc	3	LC	12	NL	Taylor (1936)
* Plestiodon lynxe* (Wiegmann, 1834)	E	SMOc	2, 3, 4	LC	10	Pr	Parker (1960)
* Plestiodon obsoletus* Baird & Girard, 1852	NE	SMOr	6	LC	11	NL	This study
FAMILY TEIIDAE							
* Aspidoscelis costatus* (Cope, 1878)	E	SMOc	3	LC	11	Pr	Zweifel (1959)
* Aspidoscelis gularis* (Baird & Girard, 1852)	NE	TVB, SMOc, CP, SMOr	1, 2, 3, 4, 5, 6	LC	9	NL	Duellman and Zweifel (1962)
* Aspidoscelis inornatus* (Baird, 1859)	NE	SMOr	5, 6	LC	14	NL	Williams (1968)
FAMILY XANTUSIIDAE							
* Xantusia extorris* Webb, 1965	E	SMOr	5	LC	15	NL	Baker et al. (1981)
* Xantusia sanchezi* Bezy & Flores-Villela, 1999	E	SMOc	3	LC	16	P	Bezy and Flores-Villela (1999)
ORDER SQUAMATA							
SUBORDER SERPENTES							
FAMILY BOIDAE							
* Boa sigma* (Smith, 1943)	E	SMOc	3	NE	15	NL	Baker et al. (1967)
FAMILY COLUBRIDAE							
* Arizona elegans* Kennicott, 1859	NE	SMOc, CP, SMOr	4, 5, 6, 7	LC	5	NL	Fleet and Dixon (1971)
* Conopsis lineata* (Kennicott, 1859)	E	CP	4, 5	LC	13	NL	Carbajal-Márquez et al. (2012)
* Conopsis nasus* (Günther, 1858)	E	SMOc, CP	2, 3, 4, 5	LC	11	NL	Taylor and Smith (1942)
* Drymarchon melanurus* (Duméril, Bibron & Duméril, 1854)	NE	SMOc	2, 3, 4	LC	6	NL	Ahumada-Carrillo y Vázquez-Huizar (2012a)
* Gyalopion canum* (Cope, 1860)	NE	CP, SMOr	5, 6	LC	9	NL	McCoy (1961)
* Lampropeltis greeri* Webb, 1961	E	SMOc	2, 4	NE	NL	NL	Liner and Dundee (1977)
* Lampropeltis polyzona* Cope, 1860	E	SMOc	2, 3	LC	11	NL	Smith and Taylor (1945)
* Lampropeltis splendida* (Baird & Girard, 1853)	NE	CP, SMOr	4, 5	LC	NL	NL	Blaney (1977)
* Leptophis diplotropis* (Günther, 1872)	E	SMOc	3, 4	LC	14	A	This study
* Masticophis bilineatus* Jan, 1863	NE	SMOc	2, 3, 4	LC	11	NL	Smith and Taylor (1945)
* Masticophis flagellum* (Shaw, 1802)	NE	SMOc, CP, SMOr	4, 5, 6, 7	LC	8	A	Conant (1965)
* Masticophis lineatus* Bocourt, 1890	E	TVB, SMOc	1,2,3,4	NE	NL	NL	Johnson (1977)
* Masticophis taeniatus* (Hallowell, 1852)	NE	SMOc, CP, SMOr	4, 5, 6	LC	10	NL	Smith and Taylor (1945)
* Mastigodryas cliftoni* (Hardy, 1964)	E	SMOc	2, 3	DD	14	NL	Villa et al. (2011)
* Oxybelis microphthalmus* Barbour & Amaral, 1926	NE	SMOc	2, 3	NE	11	NL	Keiser (1969)
* Pantherophis emoryi* (Baird & Girard, 1853)	NE	CP, SMOr	4, 5	LC	13	NL	This study
* Pituophis catenifer* (Blainville, 1835)	NE	CP, SMOr	4, 5, 6, 7	LC	9	NL	Conant (1965)
* Pituophis deppei* (Duméril, 1853)	E	TVB, SMOc, CP, SMOr	1, 2, 3, 4, 5, 6, 7, 8	LC	14	A	Zweifel (1954)
* Rhinocheilus lecontei* (Baird & Girard, 1853)	NE	SMOc, CP, SMOr	2, 3, 4, 5	LC	8	NL	Frost and Aird (1978)
* Salvadora bairdi* Jan, 1860	E	SMOc	2, 3, 4	LC	15	Pr	Smith and Taylor (1945)
* Salvadora lineata* Schmidt, 1940	NE	SMOc, CP, SMOr	4, 5, 6, 7	NE	NL	NL	Campos-Rodríguez et al. (2017)
* Salvadora mexicana* (Duméril, Bibron & Duméril, 1854)	E	SMOc	3	LC	15	Pr	Bañuelos-Alamillo et al. (2019)
* Senticolis triaspis* (Cope, 1866)	NE	TVB, SMOc	1, 2, 3, 4	LC	6	NL	Smith and Taylor (1945)
* Sonora mutabilis* Stickel, 1943	E	SMOc	3	LC	14	NL	Smith and Taylor (1945)
* Sonora semiannulata* Baird & Girard, 1853	NE	SMOr	6	LC	5	NL	This study
* Sympholis lippiens* Cope, 1862	E	SMOr	3	DD	14	NL	This study
* Tantilla atriceps* (Günther, 1895)	NE	CP, SMOr	5	LC	11	A	Cole and Hardy (1981)
* Tantilla bocourti* (Günther, 1895)	E	SMOc	2, 3, 4	LC	9	NL	McCranie (1977)
* Tantilla wilcoxi* Stejneger, 1902	NE	SMOc, CP	4, 5	LC	10	NL	McCoy (1964)
* Trimorphodon paucimaculatus* Taylor, 1938	E	SMOc	3	NE	15	NL	Bañuelos-Alamillo et al. (2015)
* Trimorphodon tau* Cope, 1870	E	SMOc	2, 3, 4	LC	13	NL	Smith and Taylor (1945)
FAMILY DIPSADIDAE							
* Diadophis punctatus* (Linnaeus, 1766)	NE	SMOc, SMOr	2, 4, 5, 6, 7	LC	4	NL	Rodríguez-Maturino et al. (2018)
* Geophis dugesii* Bocourt, 1883	E	SMOc	2, 4	LC	13	NL	García-Balderas and Quintero-Díaz (2012)
* Heterodon kennerlyi* Kennicott, 1860	NE	CP	4, 5	NE	11	NL	Smith and Taylor (1945)
* Hypsiglena affinis* Boulenger, 1894	E	SMOc	3	NE	14	Pr	Boulenger (1894)
* Hypsiglena jani* Dugès, 1865	NE	SMOc, CP, SMOr	4, 5, 6, 7	LC	6	Pr	Morafka (1977)
* Imantodes gemmistratus* (Cope, 1861)	NE	SMOc	3	LC	6	Pr	Bañuelos-Alamillo et al. (2017b)
* Leptodeira splendida* Günther, 1885	E	SMOc	2, 3	LC	14	NL	Ahumada-Carrillo et al. (2011)
* Rhadinaea hesperia* Bailey, 1940	E	SMOc	3	LC	10	Pr	Myers (1974)
* Rhadinaea laureata* (Günther, 1868)	E	SMOc	2, 3	LC	12	NL	Webb (1982)
FAMILY ELAPIDAE							
* Micruroides euryxanthus* (Kennicott, 1860)	NE	SMOc	3	LC	15	NL	Gámez-Gallegos et al. 2024
* Micrurus distans* Kennicott, 1860	E	SMOc	2, 3	LC	14	Pr	Ahumada-Carrillo and Vázquez-Huizar (2012b)
FAMILY LEPTOTYPHLOPIDAE							
* Rena dulcis* Baird & Girard, 1853	NE	SMOr	5	LC	13	NL	Flores-Villela et al. (2022)
* Rena dugesii* (Bocourt, 1881)	E	SMOc	3	NE	NL	NL	This study
FAMILYY NATRICIDAE							
* Nerodia erythrogaster* (Forster, 1771)	NE	CP	4	LC	11	A	Conant (1963a)
* Storeria storerioides* (Cope, 1866)	E	SMOc	2, 3, 4	LC	11	NL	Webb (1982)
* Thamnophis cyrtopsis* (Kennicott, 1860)	NE	TVB, SMOc, CP, SMOr	1, 2, 3, 4, 5, 6, 7, 8	LC	7	A	Webb (1980)
* Thamnophis eques* (Reuss, 1834)	NE	TVB, SMOc, CP	1, 2, 3, 4, 5	LC	8	A	Smith and Taylor (1945)
* Thamnophis errans* (Smith, 1942)	E	SMOc	2	LC	16	NL	Webb (1976)
* Thamnophis marcianus* (Baird & Girard, 1853)	NE	CP, SMOr	5, 6, 7, 8	LC	10	A	Rossman (1971)
* Thamnophis melanogaster* (Peters, 1864)	E	SMOc, CP	4	EN	15	A	Conant (1963b)
* Thamnophis pulchrilatus* (Cope, 1885)	E	SMOc	2, 3	LC	15	NL	Webb (1980)
* Thamnophis scaliger* (Jan, 1863)	E	SMOc, CP	2, 4	VU	15	A	Sigala-Rodríguez et al. (2020b)
FAMILY TYPHLOPIDAE							
* Indotyphlops braminus* (Daudin, 1803)	IN	SMOc, SMOr	4	IN	IN	IN	Bañuelos-Alamillo and Carbajal-Márquez (2016b)
FAMILY VIPERIDAE							
* Crotalus aquilus* Klauber, 1952	E	TVB, SMOc	1, 2, 4	LC	16	Pr	Carbajal-Márquez et al. (2015)
* Crotalus atrox* Baird & Girard, 1853	NE	CP, SMOr	4, 5, 6	LC	9	Pr	Dugès (1869)
* Crotalus basiliscus* (Cope, 186 4)	E	SMOc	2, 3	LC	16	Pr	Ahumada-Carrillo et al. (2011)
* Crotalus lepidus* (Kennicott, 1861)	NE	SMOc, CP, SMOr	2, 3, 4, 5	LC	12	Pr	Gloyd (1940)
* Crotalus molossus* Baird & Girard, 1853	NE	TVB, SMOc, CP, SMOr	1, 2, 3, 4, 5, 6, 7, 8	LC	8	Pr	Gloyd (1936)
* Crotalus ornatus* (Hallowell, 1854)	NE	SMOr	6	NE	16	NL	Villalobos-Juárez et al. (2025)
* Crotalus polystictus* (Cope, 1865)	E	TVB, SMOc	2, 3, 4	LC	16	Pr	Gloyd (1940)
* Crotalus pricei* Van Denburgh, 1895	NE	SMOc	2	LC	14	Pr	Sigala-Rodríguez et al. (2020b)
* Crotalus scutulatus* (Kennicott, 1861)	NE	TVB, SMOc, CP, SMOr	1, 2, 4, 5, 6	LC	11	Pr	Smith and Taylor (1945)
* Crotalus willardi* Meek, 1905	NE	SMOc	2, 4	LC	13	Pr	Smith and Taylor (1945)

Native species represent 30 families (Table 2): nine of amphibians (two of salamanders and seven anurans) and 21 of reptiles (3 of turtles, 11 of lizards and 7 of snakes). Among amphibians, most species are in the Bufonidae (6), Hylidae and Ranidae (5 each) families. Whitin reptiles, most species are in the Phrynosomatidae (32) and Colubridae (31) families. Sixty-nine native herpetofaunal genera are represented in Zacatecas: 14 of amphibians (2 of salamanders and 12 of anurans) and 55 of reptiles (3 of turtles, 19 of lizards and 33 of snakes) (Table 1). Regarding native amphibians, the most diverse genera are *Anaxyrus* and *Lithobates* with four species each. Among native reptiles, the most diverse genera are *Sceloporus* (24), *Crotalus* (10) and *Thamnophis* (7). It is important to mention that Zacatecas is the type locality of *Craugastor occidentalis*, *Sceloporus minor*, *Xantusia sanchezi* and *Hypsiglena affinis*.

**Table 2.** Summary of species present in Zacatecas by Family, Order or Suborder, and Class. Abbreviations: GD = Global Distribution: E = Endemic to Mexico; NE = No endemic to Mexico; IN = Introduced. PP = Physiographic Province: TVB = Trans-Mexican Volcanic Belt; SMOc = Sierra Madre Occidental; CP = Central Plateau; SMOr = Sierra Madre Oriental. ECOR = Ecoregions: 1 = Plains of the Trans-Mexican Volcanic Belt; 2 = Elevations of the Sierra Madre Occidental; 3 = Canyons of the Sierra Madre Occidental; 4 = Foothills and Plains of the Sierra Madre Occidental and the Central Plateau; 5 = Plains of the Zacatecano-Potosino Plateau; 6 = Low Sierras of the Southern Chihuahua Desert; 7 = Isolated elevations of the Zacatecano-Potosino Plateau; 8 = Plains in the central region of the Chihuahuan Desert. IUCN Status: DD = Data Deficient; LC = Least Concern; VU = Vulnerable; NT = Near Threatened; EN = Endangered; CR = Critically Endangered; NE = Not Evaluated (IUCN 2025). EVS = Environmental Vulnerability Score (the higher the score the greater the vulnerability): NL = Not Listed; low (L) vulnerability species (EVS of 3–9); medium (M) vulnerability species (EVS of 10–13); high (H) vulnerability species (EVS of 14–20) (Wilson et al. 2013a, 2013b). Conservation status in Mexico according to SEMARNAT (2010): NL = Not Listed; Pr = Subject to Special Protection; P = In Danger of Extinction; A = Threatened.

Taxon	genera	species	GD	PP	ECOR	IUCN	EVS	SEMARNAT
			E, NE, IN	TVB, SMOc, CP, SMOr	1, 2, 3, 4, 5, 6, 7, 8	NE, DD, LC, VU, NT, EN, CR	NL, Low, Medium, High	NL, Pr, A, P
Class Amphibia								
Order Caudata	2	3	3, 0, 0	0, 3, 1, 0	0, 3, 0, 2, 0, 1, 0, 0	0, 0, 3, 0, 0, 0, 0	0, 0, 2, 1	0, 2, 1, 0
Ambystomatidae	1	2	2, 0, 0	0, 2, 1, 0	0, 2, 0, 2, 0, 1, 0, 0	0, 0, 2, 0, 0, 0, 0	0, 0, 1, 1	0, 2, 0, 0
Plethodontidae	1	1	1, 0, 0	0, 1, 0, 0	0, 1, 0, 0, 0, 0 ,0 ,0	0, 0, 1, 0, 0, 0, 0	0, 0, 1, 0	0, 0, 1, 0
Order Anura	13	23	10, 12, 1	8, 19, 10, 7	6, 14, 13, 13, 6, 4, 2, 0	1, 0, 20, 0, 0, 1, 0	1, 11, 7, 3	18, 3, 1, 0
Bufonidae	3	6	2, 4, 0	1, 4, 4, 3	2, 3, 3, 5, 3, 2, 1, 0	0, 0, 6, 0, 0, 0, 0	0, 4, 1, 1	5, 1, 0, 0
Craugastoridae	1	2	1, 1, 0	0, 2, 0, 1	0, 2, 1, 0, 0, 1, 1, 0	0, 0, 2, 0, 0, 0, 0	0, 1, 1, 0	2, 0, 0, 0
Eleuthherodactylidae	1	2	1, 1, 0	0, 1, 1, 0	0, 1, 0, 1, 1, 0, 0, 0	1, 0, 1, 0, 0, 0, 0	1, 0, 1, 0	2, 0, 0, 0
Hylidae	3	5	3, 2, 0	3, 4, 1, 0	1, 3, 3, 2, 0, 0, 0, 0	0, 0, 4, 0, 0, 1, 0	0, 2, 2, 1	4, 0, 1, 0
Microhylidae	1	1	0, 1, 0	1, 1, 0, 0	0, 1, 1, 1, 0, 0, 0, 0	0, 0, 1, 0, 0, 0, 0	0, 1, 0, 0	1, 0, 0, 0
Ranidae	2	5	3, 1, 1	2, 5, 2, 1	2, 3, 4, 2, 0, 0, 0, 0	0, 0, 4, 0, 0, 0, 0	0, 1, 2, 1	2, 2, 0, 0
Scaphiopodidae	2	2	0, 2, 0	1, 2, 2, 2	1, 1, 1, 2, 2, 1, 0, 0	0, 0, 2, 0, 0, 0, 0	0, 2, 0, 0	2, 0, 0, 0
Subtotal amphibians:	15	26	13, 12, 1	8, 22, 11, 7	6, 17, 13, 15, 6, 5, 2, 0	1, 0, 23, 0, 0, 1, 0	1, 11, 9, 4	18, 5, 2, 0
Class Reptilia								
Order Testudines	4	5	2, 2, 1	0, 4, 1, 2	1, 1, 2, 3, 2, 2, 0, 0	0, 1, 3, 0, 0, 0, 0	0, 0, 2, 2	0, 3, 1, 0
Emydidae	2	2	1, 0, 1	0, 2, 0, 0	0, 0, 1, 1, 0, 0, 0, 0	0, 1, 0, 0, 0, 0, 0	0, 0, 0, 1	0, 1, 0, 0
Kinosternidae	1	2	1, 1, 0	0, 2, 1, 1	1, 1, 1, 2, 1, 1, 0, 0	0, 0, 2, 0, 0, 0, 0	0, 0, 2, 0	0, 2, 0, 0
Testudinae	1	1	0, 1, 0	0, 0, 0, 1	0, 0, 0, 0, 1, 1, 0, 0	0, 0, 1, 0, 0, 0, 0	0, 0, 0, 1	0, 0, 1, 0
Order Squamata								
Suborder Lacertilia	20	53	31, 20, 2	1, 30, 16, 28	1, 19, 16, 23, 22, 18, 5, 0	11, 1, 36, 1, 1, 1, 0	4, 2, 25, 20	37, 7, 6, 1
Anguidae	3	3	1, 2, 0	0, 3, 0, 1	0, 2, 0, 2, 1, 1, 0, 0	1, 0, 2, 0, 0, 0, 0	0, 0, 2, 1	2, 1, 0, 0
Anolidae	1	1	1, 0, 0	0, 1, 0, 1	0, 1, 1, 1, 0, 0, 0, 0	0, 0, 1, 0, 0, 0, 0	0, 0, 1, 0	1, 0, 0, 0
Crotaphytidae	2	2	0, 2, 0	0, 0, 0, 2	0, 0, 0, 0, 2, 2, 0, 0	0, 0, 2, 0, 0, 0, 0	0, 0, 2, 0	0, 1, 1, 0
Eublepharidae	1	1	0, 1, 0	0, 0, 0, 1	0, 0, 0, 0, 0, 1, 0, 0	0, 0, 1, 0, 0, 0, 0	0, 0, 0, 1	0, 1, 0, 0
Gekkonidae	1	2	0, 0, 2	0, 2, 1, 1	0, 0, 0, 2, 1, 1, 0, 0	0, 0, 0, 0, 0, 0, 0	0, 0, 0, 0	0, 0, 0, 0
Helodermatidae	1	1	1, 0, 0	0, 1, 0, 0	0, 0, 1, 0, 0, 0, 0, 0	0, 0, 1, 0, 0, 0, 0	0, 0, 1, 0	0, 0, 1, 0
Iguanidae	1	1	1, 0, 0	0, 1, 0, 0	0, 0, 1, 0, 0, 0, 0, 0	0, 0, 1, 0, 0, 0, 0	0, 0, 0, 1	0, 0, 1, 0
Phrynosomatidae	6	32	20, 12, 0	0, 15, 14, 18	0, 13, 7, 16, 15, 10, 5, 0	9, 1, 19, 1, 1, 1, 0	4, 1, 14, 13	27, 2, 3, 0
Phyllodactylidae	1	1	1, 0, 0	0, 1, 0, 0	0, 0, 1, 0, 0, 0, 0, 0	0, 0, 1, 0, 0, 0, 0	0, 0, 0, 1	1, 0, 0, 0
Scincidae	1	4	3, 1, 0	0, 3, 0, 1	0, 2, 2, 1, 0, 1, 0, 0	1, 0, 3, 0, 0, 0, 0	0, 0, 4, 0	3, 1, 0, 0
Teiidae	1	3	1, 2, 0	1, 2, 1, 2	1, 1, 2, 1, 2, 2, 0, 0	0, 0, 3, 0, 0, 0, 0	0, 1, 1, 1	2, 1, 0, 0
Xantusiidae	1	2	2, 0, 0	0, 1, 0, 1	0, 0, 1, 0, 1, 0, 0, 0	0, 0, 2, 0, 0, 0, 0	0, 0, 0, 2	1, 0, 0, 1
Suborder Serpentes	34	65	31, 33, 1	9, 51, 26, 24	8, 33, 35, 39, 25, 16, 10, 4	10, 2, 50, 1, 0, 1, 0	5, 16, 21, 22	38, 16, 10, 0
Boidae	1	1	1, 0, 0	0, 1, 0, 0	0, 0, 1, 0, 0, 0, 0, 0	1, 0, 0, 0, 0, 0, 0	0, 0, 0, 1	1, 0, 0, 0
Colubridae	18	31	15, 16, 0	2, 23, 14, 13	3, 14, 18, 21, 14, 8, 5, 1	5, 2, 24, 0, 0, 0, 0	4, 9, 10, 8	25, 2, 4, 0
Dipsadidae	7	9	5, 4, 0	0, 8, 2, 2	0, 4, 5, 4, 3, 2, 2, 0	2, 0, 7, 0, 0, 0, 0	0, 3, 4, 2	5, 4, 0, 0
Elapidae	2	2	1, 1, 0	0, 2, 0, 0	0, 1, 2, 0, 0, 0, 0, 0	0, 0, 2, 0, 0, 0, 0	0, 0, 0, 2	1, 1, 0, 0
Leptotyphlopidae	1	2	1, 1, 0	0, 1, 0, 1	0, 0, 1, 0, 1, 0, 0, 0	1, 0, 1, 0, 0, 0, 0	1, 0, 1, 0	2, 0, 0, 0
Natricidae	3	9	5, 4, 0	2, 7, 6, 2	2, 6, 4, 6, 3, 2, 2, 2	0, 0, 7, 1, 0, 1, 0	0, 2, 3, 4	3, 0, 6, 0
Typhlopidae	1	1	0, 0, 1	0, 1, 0, 1	0, 0, 0, 1, 0, 0, 0, 0	0, 0, 0, 0, 0, 0, 0	0, 0, 0, 0	0, 0, 0, 0
Viperidae	1	10	3, 7, 0	4, 8, 4, 5	3, 8, 4, 7, 4, 4, 1, 1	1, 0, 9, 0, 0, 0, 0	0, 2, 3, 5	1, 9, 0, 0
Subtotal reptiles	58	123	64, 55, 4	10, 85, 43, 54	10, 53, 53, 65, 49, 36, 15, 4	21, 4, 89, 2, 1, 2, 0	9, 18, 48, 44	75, 26, 17, 1
Total amphibians and reptiles	73	149	77, 67, 5	18, 107, 54, 61	16, 70, 66, 80, 55, 41, 17, 4	22, 4, 112, 2, 1, 3, 0	10, 29, 57, 48	93, 31, 19, 1

Sixteen species represent new state contributions for Zacatecas: the Spotted Chirping Frog (*Eleutherodactylus guttilatus*), the Common Bullfrog (*A. catesbeiana*), the Texas Tortoise (*Gopherus berlandieri*), the Longnose Leopard Lizard (*Gambelia wislizenii*), the Mediterranean House Gecko (*H. turcicus*), the White-bellied Rough Lizard (*Sceloporus albiventris*), the Twin-spotted Spiny Lizard (*Sceloporus bimaculosus*), the Ornate Spiny Lizard (*Sceloporus ornatus*), the Texas Spiny Lizard (*Sceloporus olivaceus*), the Bunch Grass Lizard (*Sceloporus cf. scalaris*), the Great Plains Skink (*Plestiodon obsoletus*), the Pacific Coast Parrot Snake (*Leptophis diplotropis*), the Great Plains Rat Snake (*Pantherophis emoryi*), the Ground Snake (*Sonora semiannulata*), the Mexican Short-tail Snake (*Sympholis lippiens*), and the Duges’ Threadsnake (*Rena dugesii*) (Table 1). For complete metadata of new contributions see Suppl. material 3.

In addition, 232 records belonging to 79 species represent new municipality records for Zacatecas, so their distribution in the state is updated (see Suppl. material 3). Among these municipal records, extensive distribution expansions and new ecoregions occupied within Zacatecas several species stand out, like *Ambystoma velasci*, *Anaxyrus compactilis*, *Lithobates magnaocularis*, *Kinosternon integrum*, *Elgaria kingii*, *Drymarchon melanurus*, *Lampropeltis splendida*, *Masticophis taeniatus*, *Oxybelis microphthalmus*, *Senticolis triaspis*, and *Tantilla wilcoxi* (see Suppl. material 3).

### General distribution

Seventy-seven (53.5%) of the 144 native species of amphibians and reptiles that inhabit Zacatecas are endemic to Mexico. Although none is a state endemic, the state harbors populations of many regional endemics that only have small populations in north-central Mexico like *Smilisca dentata*, *Sceloporus aurantius*, *S. ornatus*, *S. huichol*, *S. shannonorum*, *Xantusia extorris*, *X. sanchezi*, *Lampropeltis greeri*, *Sonora mutabilis*, *H. affinis*, and *R. dugesii*. The remaining 67 of the species of amphibians and reptiles are not endemic to Mexico, out of which 59 are shared with the United States of America (nine frogs, two turtles, 20 lizards and 28 snakes), one with Central American countries (1 snake), and six with both United States of America and Central American countries (2 frogs and 4 snakes). Vast and important areas of Zacatecas remain poorly sampled due to the difficulty in accessing them, where more native amphibian and reptile species potentially inhabit. Therefore, we suggest 35 potential species, 13 amphibians and 22 reptiles, that could be documented for Zacatecas once more work is done in the state (Table 3).

**Table 3.** List of amphibian and reptile species that potentially occur in Zacatecas, Mexico.

Taxon	Potential occurrence
Amphibians	
* Ambystoma silvense* Webb, 2004	Western Zacatecas
* Anaxyrus mexicanus* (Brocchi, 1879)	Western Zacatecas
* Anaxyrus speciosus* (Girard, 1854)	Northeastern Zacatecas
* Incilius mazatlanensis* (Taylor, 1940)	Western Zacatecas
* Incilius mccoyi* (Santos-Barrera & Flores-Villela, 2011)	Western Zacatecas
* Eleutherodactylus pallidus* (Duellman, 1958)	Southwestern Zacatecas
* Eleutherodactylus verrucipes* (Cope, 1885)	Eastern Zacatecas
* Eleutherodactylus wixarika* Reyes-Velasco, Ahumada-Carrillo, Burkhardt & Devitt, 2015	Western Zacatecas
* Exerodonta smaragdina* (Taylor, 1940)	Southwestern Zacatecas
* Smilisca baudinii* (Duméril & Bibron, 1841)	Southwestern Zacatecas
* Tlalocohyla smithi* (Boulenger, 1902)	Southwestern Zacatecas
* Lithobates forreri* (Boulenger, 1883), 2022)	Southwestern Zacatecas
* Lithobates pustulosus* (Boulenger, 1883)	Southwestern Zacatecas
Reptiles	
* Iguana iguana* (Linnaeus, 1758)	Southwestern Zacatecas
* Sceloporus nelsoni* Cochran, 1923	Southwestern Zacatecas
* Sceloporus samcolemani* Smith & Hall, 1974	Northeastern Zacatecas
* Uma exsul* Schmidt & Bogert, 1947	Northern Zacatecas
* Plestiodon dicei* (Ruthven & Gaige, 1933)	Northeastern Zacatecas
* Plestiodon parviauriculatus* (Taylor, 1933)	Northwestern Zacatecas
* Aspidoscelis marmoratus* (Baird & Girard, 1852)	Northern Zacatecas
* Xantusia bolsonae* Webb, 1970	Northern Zacatecas
* Bogertophis subocularis* (Brown, 1901)	Northern Zacatecas
* Coniophanes lateritius* Cope, 1862	Southwestern Zacatecas
* Enulius oligostichus* Smith, Arndt & Sherbrook, 1967	Southwestern Zacatecas
* Lampropeltis alterna* (Brown, 1901)	Northeastern Zacatecas
* Lampropeltis leonis* (Günther, 1893)	Northeastern Zacatecas
* Lampropeltis mexicana* (Garman, 1884)	Southeastern Zacatecas
* Masticophis schotti* Baird & Girard, 1853	Eastern Zacatecas
* Tantilla hobartsmithi* Taylor, 1937	Northern Zacatecas
* Tantilla nigriceps* Kennicott, 1860	Northern Zacatecas
* Manolepis putnami* (Jan, 1863)	Southwestern Zacatecas
* Rena klauberi* Flores-Villela, Smith, Canseco-Márquez & Campbell, 2022	Southwestern Zacatecas
* Rena segrega* (Klauber, 1939)	Northern Zacatecas
* Storeria dekayi* (Holbrook, 1839)	Northeastern Zacatecas
* Agkistrodon bilineatus* Günther, 1863	Southwestern Zacatecas

### Conservation status

One hundred and twenty-two native species of Zacatecas (24 amphibians and 98 reptiles) are listed by the IUCN (2025). Out of these, one amphibian (*S. dentata*) and two reptiles (*Sceloporus goldmani* and *Thamnophis melanogaster*) are considered Endangered (EN), two of the reptiles are Vulnerable (VU) (*Sceloporus maculosus* and *Thamnophis scaliger*), one reptile is Near Threatened (NT) *Sceloporus ornatus*, 23 amphibians and 89 reptiles are Least Concern (LC), four reptiles are Data Deficient (DD) (*Terrapene nelsoni*, *S. shannonorum*, *Mastigodryas cliftoni* and *Sympholis lippiens*), and one amphibian and 21 reptiles are not evaluated (NE) (Tables 1, 2).

According to the EVS, 134 native species to Zacatecas are considered to occur (24 amphibians and 110 reptiles). Out of these, four amphibians (*A. rosaceum*, *A. compactilis*, *S. dentata* and *Lithobates psilonota*) and 44 reptiles (e.g., *Terrapene nelsoni*, *S. aurantius*, and *Thamnophis errans*) are considered of high vulnerability; nine amphibians (e.g., *C. occidentalis* and *Agalychnis dacnicolor*) and 48 reptiles (e.g., *K. integrum*, *Crotaphytus collaris*, and *Geophis dugesii*) are considered of medium vulnerability; 11 amphibians (e.g., *Anaxyrus cognatus* and *Craugastor augusti*) and 18 reptiles (e.g., *Sceloporus grammicus* and *Crotalus atrox*) are considered of low vulnerability; and one amphibian and nine reptiles are not evaluated (Tables 1, 2).

Finally, in SEMARNAT (2010), 51 native species (seven amphibians and 44 reptiles) are in some risk category. Out of these, one reptile is in Danger of Extinction (P) (*X. sanchezi*), two amphibians (*Isthmura bellii* and *S. dentata*) and 17 reptiles (e.g., *G. berlandieri*, *Heloderma horridum*, and *L. diplotropis*) are Threatened (A), five amphibians (*Ambystoma rosaceum*, *A. velasci*, *Anaxyrus debilis*, *Lithobates berlandieri* and *L. montezumae*) and 26 reptiles (e.g., *K. integrum*, *G. wislizenii*, and *Crotalus pricei*) are Subject to Special Protection (Pr), and 18 amphibians and 75 reptiles are not listed (Tables 1, 2).

### Patterns of physiographic distribution

We recognize four physiographic provinces in Zacatecas (Fig. 2) and eight ecoregions (Fig. 3). The distribution of the herpetofauna of Zacatecas among these regions is documented in Table 1 and summarized in Table 2. The numbers of species in the four physiographic provinces ranges from a low in TVB (18), medium in CP (54) and SMOr (61), to high in SMOc (107). The numbers of species were different for amphibians and reptiles found in the SMOc (22/85), CP (11/43), SMOr (7/54), and only similar to those found in TVB (8/10). The percentage of the herpetofaunal species present in each of the four physiographic provinces are TVB (12.1%), CP (36.2%), SMOr (41.0%) and SMOc (72.0%). The mean percentage of occupancy is 40.3%.

The number of species of herpetofauna found in the eight ecoregions (Fig. 3) ranges from low in 8(4), 1(16) and 7(17), medium in 6(41) and 5(55), to high in 3(66), 2(70) and 4(80). The percentage of the herpetofaunal species in each ecoregion, in order of size, are ecoregion 8(2.7%), 1(10.7%), 7(11.4%), 6(27.5%), 5(37.0%), 3(44.3%), 2(47.0%), and 4(53.7%). The mean percentage of ecoregions occupancy is 29.3%. The numbers of species among the two major groups (amphibians and reptiles) found in each ecoregion were different in all: 1(6/10), 2(17/53), 3(13/53), 4(15/65), 5(6/49), 6(5/36), 7(2/15), 8(0/4).

### Comparisons to the herpetofaunas of adjacent states

Zacatecas is a state in north central Mexico bordered mainly by Aguascalientes, Coahuila, Durango, Jalisco, San Luis Potosí, and barely by Guanajuato, Nayarit, and Nuevo León (Fig. 1). The lists of herpetofauna of these states have been recently updated. The herpetofauna of Aguascalientes has been studied by Carbajal-Márquez and Quintero-Díaz (2016), Coahuila by Lemos-Espinal and Smith (2016), Durango by Lemos-Espinal et al. (2019), Guanajuato by Leyte-Manrique et al. (2022), Jalisco by Cruz-Sáenz et al. (2017), Nayarit by Woolrich-Piña et al. (2016), Nuevo León by Nevárez-De los Reyes et al. (2016), and San Luis Potosí by Lemos-Espinal et al. (2018).

We compare the herpetofauna of Zacatecas with neighboring states indicating the total number of species, endemic species, non-endemic, non-native species, and proportion of endemic species (Table 4). The number of herpetofaunal species per state ranges from a minimum of 90 in Aguascalientes to a maximum of 223 in Jalisco. The number of Mexican endemics ranges from the lowest in Coahuila (35) to the highest in Jalisco (144). The proportion of endemism ranges from 26.3% in Coahuila to 64.6% in Jalisco. The number of non-endemics ranges from a minimum of 40 in Aguascalientes to a maximum of 105 in San Luis Potosí. Finally, the number of non-native species ranges from one in Coahuila to six in Aguascalientes. Within these nine states, Zacatecas is the fourth with the largest territorial extension, and is the fifth richest in herpetofauna, the third in country endemic species (77) and the fourth in proportion of endemism (51.7%). It is also above the average proportion of endemism for the nine states (46.5%). Additionally, Zacatecas is the fourth with the lowest number of non-endemic species (67) and shares second place with Guanajuato for the highest number of non-native species (five).

**Table 4.** Comparison of the number of total herpetofauna, endemic, non-endemic, non-native species, the percentage of endemism, and total state area for Zacatecas, Mexico, and adjoining states.

State	Total herpetofauna	Endemics	% Endemism	Non endemics	Non-native	Area km^2^
Zacatecas (current study)	149	77	51.7	67	5	75,275.3
Aguascalientes Carbajal-Márquez and Quintero-Díaz (2016)	90	44	48.9	40	6	5,615.7
Coahuila Lemos-Espinal and Smith (2016)	133	35	26.3	97	1	151,594.8
Durango Lemos-Espinal et al. (2019)	157	73	46.5	81	3	123,364.0
Guanajuato Leyte-Manrique et al. (2022)	101	56	55.4	40	5	30,606.7
Jalisco Cruz-Sáenz et al. (2017)	223	144	64.6	75	4	78,595.9
Nayarit Woolrich-Piña et al. (2016)	154	88	57.1	61	4	27, 856.5
Nuevo Léon Nevárez-De los Reyes et al. (2016)	139	39	28	96	4	64,156.2
San Luis Potosí Lemos-Espinal et al. (2018)	182	73	40.1	105	4	61,138.0

## Discussion

In this work, we report 149 species of herpetofauna for Zacatecas, that is, 16 species more than those reported by Sigala-Rodríguez et al. (2020a, 2020b), and 31 species more than the latest list recently published by Ramírez-Bautista et al. (2023), demonstrating that fieldwork is essential, complemented by the review of literature, museums, and databases to gain knowledge about the herpetofauna of a state. In fact, of the 16 new records for Zacatecas, 12 belong to our fieldwork and four belong to the review of collection records. Zacatecas is one of the least studied states on biodiversity with other states in the north-central part of the country, which has created the false perception that it is not biologically diverse (Hernández-Ramírez 2020). However, its extension (i.e., eighth largest state in Mexico), and its geographical location in which various ecoregions, physiographic provinces, and types of habitats converge, make it a potential diverse region, even for herpetofauna, and mainly for reptiles. Particularly, it has a high richness of lizards in the genus *Sceloporus* (24 species), which is greater than that of its neighboring states; Aguascalientes (12), Coahuila (19), Durango (19), Nuevo León (18), San Luis Potosí (14), and only surpassed by Jalisco (25) (Carbajal-Márquez and Quintero-Díaz 2016; Lemos-Espinal and Smith 2016; Nevárez-de los Reyes et al. 2016; Cruz-Sáenz et al. 2017; Lemos-Espinal et al. 2018, 2019). In addition, it must be considered that within the genus *Sceloporus*, there are several species complexes (Díaz-Cárdenas et al. 2017; Campillo-García et al. 2021), and the number of species in this genus may increase in the future.

As a result of specimens found in the field by the authors, verification of specimens in museums, and review of specialized literature, we obtained a total of 16 new state records, as well as 232 new municipal records from 79 species that had not been formally reported. In the case of the record of *S. cf. scalaris*, the review of specimens did not lead us to identify any of the species of the *S. scalaris* group reported for surrounding areas (e.g., *S. chaneyi*, *S. goldmani* or *S. samcolemani*). Therefore, our records suggest that the distribution of *S. scalaris* may extend further north than previously known, or that undescribed species may be present. Traditional morphological characters used to describe taxa within the *S. scalaris* group have been shown to exhibit strong morphological overlap and likely have limited utility (Grummer and Bryson 2014). It is noteworthy that several new state records and municipal distribution extensions have been made from recent field surveys, despite the increase in insecurity in the Zacatecas and surrounding areas due to violence generated by criminal groups which prevents access to many areas (e.g., Ahumada-Carrillo et al. 2014; Bañuelos-Alamillo and Osegueda-Berrios 2020; Carbajal-Márquez et al. 2020a, 2020b, 2020c, 2020d; Rosales-Martínez et al. 2022). Unfortunately, some authors of this study experienced security threats associated with criminal groups operating in Zacatecas, thus limiting field survey efforts. Due to these complications, several areas within the state remain inaccessible for further efforts to search for herpetofauna and biodiversity in general, and probably for this reason, an updated checklist has not been made, as well as available population data on the herpetofauna. This also suggests that the herpetofaunal richness of Zacatecas may increase as more areas become accessible (supported by our list of potential species in Table 3) and demonstrates the need for systematic sampling covering all municipalities and habitat types in the state.

### General distribution

Despite its richness in amphibians and reptiles, Zacatecas does not have state endemics. However, several regional endemics are present (e.g., *S. dentata* and *S. aurantius*) with populations found only in north-central Mexico, and thus, Zacatecas serves as a reservoir for regional endemism. Additionally, large areas of Zacatecas have not been systematically explored. It is probable that our surveys have missed identifying new state records and/or state or regional endemics. Therefore, the richness of the herpetofauna of Zacatecas may be greater than we currently understand. For regions that have received little attention, we encourage further surveys and studies. For more direct and effective sampling strategies, we recommend the available literature for the state (Lara-Galván et al. 2020, 2023).

Additionally, we report the minimum and maximum elevation records for several species enriching knowledge about their vertical distribution in meters above sea level (m a.s.l. = m). Among the amphibians, we have *Anaxyrus cognatus* with an elevation of 2,637 m, which exceeds the maximum known elevation of 2,500 m by 137 m (Lemos-Espinal et al. 2019). *Hypopachus variolosus* with an elevation of 2,234 m, exceeding the previous maximum elevation of 2,200 m by 34 m (Lemos-Espinal and Dixon 2013). *Lithobates magnaocularis* with an elevation of 1,977 m, exceeding the previous maximum elevation of 1,815 m by 162 m (Carbajal-Márquez et al. 2023).

Among reptiles, specifically turtles and lizards, we have *Gopherus berlandieri* at 2,115 m, which exceeds the previous elevation of 884 m by 1,231 m (Lemos-Espinal and Smith 2007). *Gambelia wislizenii* at 2,044 m, exceeds the previously reported maximum elevation of 1,500 m by 544 m (Heimes 2022). *Coleonyx brevis* at 2,072 m, exceeds the previously reported maximum elevation of 1,800 m by 272 m (Lemos-Espinal et al. 2019). *Sceloporus albiventris* at 2,375 m, exceeds the previous maximum elevation of 1,650 m by 725 m (Heimes 2016). *Sceloporus bimaculosus* at 2,148 m is 948 m higher than the previous maximum elevation of 1,200 m (Heimes 2022). *Sceloporus ornatus* at 2,694 m is 1,594 m higher than the previous maximum reported elevation of 1,100 m (Heimes 2022). *Sceloporus shannonorum* at 2,607 m is just higher than the previous maximum reported elevation of 2,600 m (Heimes 2022). *Aspidoscelis gularis* at 2,637 m is 637 m higher than the previous maximum elevation of 2,000 m (Lemos-Espinal et al. 2019).

Regarding snakes, *Drymarchon melanurus* at 2,086 m is 186 m above the highest reported elevation of 1,900 m (Heimes 2016). *Lampropeltis splendida* at 1,941 m is 141 m above the highest reported elevation of 1,800 m (Heimes 2016). *Rhinocheilus lecontei* at 2,024 m is 124 m above the highest reported elevation of 1,900 m (Heimes 2016). *Salvadora lineata* at 2,222 m is slightly above the previously reported elevation of 2,200 m (Heimes 2016). *Sonora semiannulata* at 2,129 m is 329 m above the previously reported elevation of 1,800 m (Heimes 2016). *Tantilla wilcoxi* at 2,620 m is 180 m higher than the previously reported 2,440 m (Heimes 2016). *Rena dugesii* at 1,578 m is slightly higher than the previously reported 1,500 m (Heimes 2016). Similarly, *Thamnophis eques* at 2,615 m is slightly higher than the previously reported 2,600 m (Heimes 2016). Finally, *Thamnophis scaliger* at 1,628 m is the lowest reported elevation, the previous one being 2,240 m, a difference of 612 m (Heimes 2016).

### Conservation status of herpetofauna of Zacatecas

We found that the native herpetofauna of Zacatecas has relatively few species of conservation concern at international and national scale (IUCN and SEMARNAT lists) but has greater conservation concerns when considering the EVS. This difference may be due to the fact that the IUCN (2025) and SEMARNAT (2010) lists are not constantly updated, so they do not take into account recent taxonomic changes, adjustments in the distribution of species, as well as the description of new species. Regarding SEMARNAT (2010), the only legally binding regulation in Mexico, it uses less rigorous and often outdated criteria. Therefore, the inclusion or exclusion of species may be subject to greater debate or political pressure, rather than being based strictly on robust scientific data. This results in an underrepresentation of species at risk compared to the IUCN or the EVS. This discrepancy between lists means that several species are not protected under Mexican legislation, which can lead to inappropriate management decisions that further endanger wild populations. Therefore, the fact that Zacatecas is home to several species of conservation concern according to EVS makes it an important state for the conservation of the regional herpetofauna.

### Patterns of physiographic distribution

In Zacatecas, the physiographic province with the highest number of herpetofauna species richness is the SMOc (39.0% of the state area), followed by the SMOr (14.8% of the state area) and with the lowest species number the CP (45.1% of the state area), and the TVB (1.1% of the state area). This is probably because the mountain ranges (SMOc and SMOr) offer a mosaic of habitats including high altitude habitats, plateaus and canyons, various vegetation types, and greater rainfall, as well as an altitudinal, thermal and humidity gradient that favors greater species richness. The opposite occurs with the CP, since it has more homogeneous environments and less precipitation, despite being the province with the largest area in the state (De la Trinidad-Cabrera 2020). Finally, the lowest species richness is registered in the TVB, despite being a mountain range, but this may be because it is the smallest province within the state. This corresponds in the same way with the richness of herpetofauna by ecoregions thanks to its affinity with each of the physiographic provinces in Zacatecas.

### Herpetofauna of adjacent states

Zacatecas shares several species of herpetofauna with its neighboring states and such overlap is not unexpected, since they share physiographic provinces and several habitats. Lemos-Espinal and Smith (2023) found greater similarity in amphibian species between Aguascalientes and Zacatecas, while for reptiles, a cluster resulted where the states of Aguascalientes, Zacatecas, Durango, Chihuahua and Sonora are grouped. Also, we found that Zacatecas is the fourth Mexican state in territorial extension within the adjoining states, as well as the fifth in richness of amphibians and reptiles. Despite this, it is the third in country endemic species presence after Jalisco and Nayarit, and above the average of the eight neighboring states in proportion of endemism, which makes Zacatecas an important reservoir for the regional herpetofauna, and a clear target to establish Natural Protected Areas (NPAs), and these may even be interstate. In addition, the information hereby presented is key to the integration and strengthening of NPAs already established and which, according to Errejón-Gómez (2023), require more information about the elements that integrate them, with their conservation status.

### Threats to the herpetofauna of Zacatecas

In Zacatecas, amphibians and reptiles face various anthropogenic threats. They are killed for medicinal, traditional, and economic purposes, out of fear or ignorance (Gamboa-Arteaga and Sigala-Rodríguez 2020), or killed by cars in the roads (Sigala-Rodríguez et al. 2020b). Regular surveys to assess population trends would help evaluate these impacts. To ensure the persistence of the herpetofaunal diversity (and biodiversity in general) it is not only necessary to protect species, but also the habitat they occupy, including those under the influence of human activities. This would serve to link communities and sustainable resource management. Globally, amphibians and reptiles face conservation threats due to the decrease in the quantity and quality of the habitats they occupy. Competition with exotic species, as well as human activities (e.g., agriculture and livestock grazing, industry, roads, urban centers, and mining) highly impact habitat quality. Global climate change is an additional threat factor, since climate is less stable than in previous decades, and erratic precipitation patterns affect these and other organisms that depend on rain for their reproduction (Farooq et al. 2024). The reasons for the decline of herpetofauna are varied, complex and inter-connected. These include habitat loss, water, soil, air pollution, excessive use of natural resources, infectious diseases, and climate change (Sigala-Rodríguez et al. 2020a).

Another issue that must be considered is public safety, which, from the perspective of green criminology, is an essential element in studies related to the environment (Carpio-Domínguez 2021). The violence generated by conflicts between criminal groups represent one of the main threats to environmental conservation in Mexico and particularly in Zacatecas. Although the presence of criminal groups in some natural areas has resulted in the reduction of destructive activities such as logging, fishing, and illegal hunting (Carpio-Domínguez 2021) and this impact might appear beneficial, it is necessary to evaluate the environmental impact of these criminal groups on species reproduction and survival. We encourage herpetofauna inventories at the municipal level to evaluate population trends of the species that inhabit Zacatecas. This would generate conservation assessments for the IUCN’s Red List and contribute to current conservation strategies for species in Zacatecas. In conclusion, this work represents a formal effort at listing the amphibians and reptiles of Zacatecas and lays the groundwork for future projects on biological, ecological, evolutionary, genetic, ethnobiological and epidemiological aspects.

## Additional information

### Artificial Intelligence (AI) use

The authors accept full responsibility for the content of the manuscript, including the disclosure of any use of AI.

No AI tools were used in the preparation of this manuscript.

### Author ORCIDs

J.J. Sigala-Rodríguez https://orcid.org/0000-0002-5895-1568

R.A. Carbajal-Márquez https://orcid.org/0000-0002-7407-0402

A. Encarnación-Luévano https://orcid.org/0000-0003-1930-375X

J.L. Lara-Galván https://orcid.org/0000-0003-0206-3673

### Data availability

All of the data that support the findings of this study are available in the main text or Supplementary Information.

## Supplementary materials

Supplementary material 1 Biological collections from which we retrieved records for each species from Zacatecas Data type docx Explanation note Here we enlist biological collections from which we retrieved records for each species from Zacatecas. https://binary.pensoft.net/file/1666601This dataset is made available under the Open Database License (http://opendatacommons.org/licenses/odbl/1.0/). The Open Database License (ODbL) is a license agreement intended to allow users to freely share, modify, and use this Dataset while maintaining this same freedom for others, provided that the original source and author(s) are credited. José Jesús Sigala-Rodríguez, Rubén Alonso Carbajal-Márquez, Alondra Encarnación-Luévano, Jesús Lenin Lara-Galván, Jorge Alberto Bañuelos-Alamillo, Eric Centenero-Alcalá, Iván Trinidad Ahumada-Carrillo, Fahd Henrry Carmona-Torres

Supplementary material 2 List of references cited in Table 1 Data type docx Explanation note Here we enlist the references cited in Table 1. https://binary.pensoft.net/file/1666602This dataset is made available under the Open Database License (http://opendatacommons.org/licenses/odbl/1.0/). The Open Database License (ODbL) is a license agreement intended to allow users to freely share, modify, and use this Dataset while maintaining this same freedom for others, provided that the original source and author(s) are credited. José Jesús Sigala-Rodríguez, Rubén Alonso Carbajal-Márquez, Alondra Encarnación-Luévano, Jesús Lenin Lara-Galván, Jorge Alberto Bañuelos-Alamillo, Eric Centenero-Alcalá, Iván Trinidad Ahumada-Carrillo, Fahd Henrry Carmona-Torres

Supplementary material 3 List of species with complete metadata that represent new state and municipality records for Zacatecas, Mexico. * = new state record Data type docx Explanation note Here we enlist species with complete metadata that represent new state and municipality records for Zacatecas, Mexico. https://binary.pensoft.net/file/1666603This dataset is made available under the Open Database License (http://opendatacommons.org/licenses/odbl/1.0/). The Open Database License (ODbL) is a license agreement intended to allow users to freely share, modify, and use this Dataset while maintaining this same freedom for others, provided that the original source and author(s) are credited. José Jesús Sigala-Rodríguez, Rubén Alonso Carbajal-Márquez, Alondra Encarnación-Luévano, Jesús Lenin Lara-Galván, Jorge Alberto Bañuelos-Alamillo, Eric Centenero-Alcalá, Iván Trinidad Ahumada-Carrillo, Fahd Henrry Carmona-Torres
