# Supplementary material for: Annotated checklist of the amphibians and reptiles of Zacatecas, Mexico
Source: Zookeys. 2026 Jun 2;1281:21–48. doi: 10.3897/zookeys.1281.174112 (PMC13250616; doi:10.3897/zookeys.1281.174112)
Supplement: Supplementary material 1 — Biological collections from which we retrieved records for each species from Zacatecas [file zookeys-1281-021_article-174112__-s001.docx]

**Appendix 1.** Biological collections from which we retrieved records for each species from Zacatecas.

**AMNH** - Collection of Herpetology, Herpetology Department, American Museum of Natural History.

**ASNHC** - Angelo State Natural History Collections, Angelo State University.

**BMNH-UM** - Herpetological Collection, Bell Museum of Natural History, University of Minnesota.

**CAS** - Collection of Herpetology, Herpetology Department, California Academy of Sciences.

**CMNH** - Amphibians and Reptiles Collection, Carnegie Museum of Natural History.

**CNAR** - Colección Nacional de Anfibios y Reptiles, Instituto de Biología UNAM.

**CZ-UAA** - Colección Zoológica, Universidad Autónoma de Aguascalientes.

**EALC** - Ernest A. Liner Personal Collection.

**ENCB** - Colección Herpetológica, Departamento de Zoología, Escuela Nacional de Ciencias Biológicas.

**FMNH** - Division of Amphibians and Reptiles, Field Museum of Natural History.

**FWMSH** - Fort Worth Museum of Sciences and History.

**LACM** - Herpetology Section, Natural History Museum of Los Angeles County.

**LSUMZ** - Collection of Amphibians and Reptiles, Louisiana State University Museum of Natural Science.

**MCZ** - Collection of Herpetology, Museum of Comparative Zoology, Harvard University Cambridge.

**MNKUH** - Museum of Natural History, Division of Herpetology, Kansas University.

**MSUM** - Herpetology Collection, Michigan State University Museum.

**MVZ** - Collection of Herpetology, Museum of Vertebrate Zoology, Division of Biological Sciences, University of California Berkeley.

**MZFC** - Colección Herpetológica, Museo de Zoología “Alfonso L. Herrera”, Facultad de Ciencias UNAM.

**NHM** - Herpetology Collections, Natural History Museum of London.

**SDNHM** - Collection of Herpetology, San Diego Natural History Museum.

**TCWC** - Collection of Herpetology, Texas Cooperative Wildlife Collection, Texas A&M University.

**TNHC** - Collection of Herpetology, Texas Natural History Collection, University of Texas Austin.

**UAZ** - Amphibians and Reptiles Collection, University of Arizona.

**UCM** - Collection of Herpetology, University of Colorado Museum.

**UF** - University of Florida Herpetology, Florida Museum of Natural History.

**UIMNH** - Collection of Herpetology, University of Illinois Museum of Natural History;

**UMMZ** - Collection of Herpetology, Museum of Zoology, University of Michigan Ann Arbor.

**USNM** - Collection of Herpetology, Department of Vertebrate Zoology, National Museum of Natural History Smithsonian Institute.

**UTADC** - University of Texas at Arlington Digital Collection = Amphibian and Reptile Diversity Research Center.

**UTAMM** - University of Texas at Arlington, Merriam Museum.

**UTEP** - Collection of Herpetology, University of Texas, El Paso.
