# Supplementary material for: Annotated checklist of the amphibians and reptiles of Zacatecas, Mexico
Source: Zookeys. 2026 Jun 2;1281:21–48. doi: 10.3897/zookeys.1281.174112 (PMC13250616; doi:10.3897/zookeys.1281.174112)
Supplement: Supplementary material 2 — List of references cited in Table 1 [file zookeys-1281-021_article-174112__-s002.docx]

**Appendix 2.** List of references cited in Table 1.

Ahumada-Carrillo IT, Vázquez-Huizar O, Vázquez-Díaz J, García-Vázquez UO (2011) Noteworthy Records of Amphibians and Reptiles from Zacatecas, México. Herpetological Review 42(3): 397–398.

Ahumada-Carrillo IT, Vázquez-Huizar O (2012a) Geographic distribution: *Drymarchon melanurus* (Central American Indigo Snake). Herpetological Review 43(3): 448.

Ahumada-Carrillo IT, Vázquez-Huizar O (2012b) Geographic distribution: *Micrurus distans* (West Mexican Coral Snake). Herpetological Review 43(1): 106.

Ahumada-Carrillo IT, Pérez-Rivera N, Reyes-Velasco J, Grünwald CI, Jones JM (2014) Notable records of amphibians and reptiles from Colima, Nayarit, Jalisco, and Zacatecas, México. Herpetological Review 45(2): 287–291.

Anderson JD, Webb RG (1978) Life History Aspects of the Mexican Salamander *Ambystoma rosaceum* (Amphibia, Urodela, Ambystomatidae). Journal of Herpetology 12(1): 89–93.

Ávila-Villegas H (2007) Geographic distribution: *Heloderma horridum horridum* (Mexican Beaded Lizard). Geographic distribution. Herpetological Review 38(2): 216.

Baker RH, Webb RG, Dalby P (1967) Notes on Reptiles and Mammals from Southern Zacatecas. American Midland Naturalist 77(1): 223–226.

Baker RH, Baker MW, Johnson JD, Webb RG (1981) New records of mammals and reptiles from northwestern Zacatecas, México. The Southwestern Naturalist 25(4): 568–569.

Ballinger RE; Tinkle, DW (1972) Systematics and evolution of the genus *Uta* (Sauria: Iguanidae). Miscellaneous publications, Museum of Zoology, University of Michigan 145: 1–83.

Bañuelos-Alamillo JA, Carbajal-Márquez RA, Quintero-Díaz GE, Moreno-Ochoa G (2015) Geographic Distribution: *Trimorphodon paucimaculatus* (Sinaloan lyresnake). Herpetological Review 46(3): 387.

Bañuelos-Alamillo JA, Carbajal-Márquez RA, Trujillo de la Torre IY (2016a) *Hemidactylus frenatus* Duméril & Bibron, 1836. Distribution notes. Mesoamerican Herpetology 3(1): 180–181.

Bañuelos-Alamillo JA, Carbajal-Márquez RA (2016b) Distribution Notes. *Indotyphlops braminus* (Daudin, 1803). Mesoamerican Herpetology 3(1): 204.

Bañuelos-Alamillo JA, Trujillo-De la Torre IY, Quintero-Díaz GE, Carbajal-Márquez RA (2017a) The Lane´s Leaf-toed Gecko, *Phyllodactylus lanei* Smith, 1935: a new state record for Zacatecas, Mexico. Herpetology Notes 10: 669–671.

Bañuelos-Alamillo JA, Trujillo-De la Torre IY, Quintero-Díaz GE, Carbajal-Márquez RA (2017b) The Central American tree snake, *Imantodes gemmistratus* (Cope, 1861) (Squamata: Dipsadinae): a new record for Zacatecas, Mexico. Check List 13(3): 2115; <https://doi.org/10.15560/13.3.2115>

Bañuelos-Alamillo JA, Ahumada-Carrillo IT, Quintero-Díaz GE, Carbajal-Márquez RA (2019) The Mexican Patch-nosed Snake, *Salvadora mexicana* (Duméril, Bibron & Duméril, 1854; Squamata: Colubridae): a new state record for Zacatecas, Mexico, and a new prey species. Cuadernos de herpetología 33(1):45–47.

Bañuelos-Alamillo JA, Osegueda-Berrios CAR (2020) La tortuga de orejas rojas (*Trachemys scripta elegans*): reptil exótico en Malpaso. In: La Biodiversidad en Zacatecas. Estudio de Estado. CONABIO, México, 383–384.

Bezy RL, Flores-Villela O (1999) A new species of *Xantusia* (Squamata: Xantusiidae) from Zacatecas, Mexico. Herpetologica 55(2): 174–184.

Blaney RM (1977) Systematics of the common kingsnake, *Lampropeltis getulus* (Linnaeus). Tulane Studies in Zoology and Botany 19(3–4): 47–103.

Boulenger GA (1894) Catalogue of the snakes in the British Museum (Natural History). Volume II. Containing the Conclusion of the Colubridae Aglyphae. British Museum (Natural History), London, xi, 382.

Boulenger, GA (1898) Fourth Report on Additions to the Batrachian Collection in the Natural-History Museum. Proceedings of the General Meeting for Scientific Business on the Zoological Society of London. Messrs, Longmans, Green, and Co., Paternoster Row, London, United Kingdom, 473–492.

Campillo-García G, Flores-Villela O, Oliver-Butler B, Velasco-Vinasco JA, Ramírez-Corona F (2021) Hidden diversity within a polytypic species: The enigmatic *Sceloporus torquatus* Wiegmann, 1828 (Reptilia, Squamata, Phrynosomatidae). Zoology 71: 781–798. <https://doi.org/10.3897/vz.71.e71995>

Campos-Rodríguez JI, Chambert L, Díaz M del C (2004) Geographical distribution: *Gerrhonotus liocephalus* (Texas Alligator Lizard). Herpetological Review 35(3): 286–287.

Campos-Rodríguez JI, Flores-Leyva X, Lorenzo-Márquez MG, Toledo-Jiménez LM (2017) New records and distribution extensions of reptiles (Reptilia: Squamata) for the state of Zacatecas, Mexico. Acta Zoológica Mexicana 33(1): 151–153.

Carbajal-Márquez RA, Arenas-Monroy JC, González-Saucedo ZY, Jones JM, Gallegos-Román L (2012) Geographic distribution: *Conopsis lineata*. (Lined Tolucan Ground Snake). Herpetological Review 43(1): 105

Carbajal-Márquez RA, González-Saucedo ZY, Arenas-Monroy JC (2015) *Crotalus aquilus* (Squamata: Viperidae), a New State Record for Zacatecas, Mexico. Acta Zoológica Mexicana 31(1): 131–133.

Carbajal-Márquez RA, Quintero-Díaz GE (2016) Poblaciones nuevas de *Sceloporus goldmani* (Squamata: Phrynosomatidae), especie considerada extinta. Revista Mexicana de Biodiversidad 87: 1395–1398. <https://doi.org/10.1016/j.rmb.2016.09.002>

Chrapliwy PS (1956) Extensions of known range of certain amphibians and reptiles of Mexico. Herpetologica 12(2): 121–124.

Cole CJ and Hardy LM (1981). Systematics of North American colubrid snakes related to *Tantilla planiceps* (Blainville). Bulletin of the American Museum of Natural History 171(3): 199284.

Conant R (1963a) Another new water snake of the genus *Natrix* from the Mexican plateau. Proceedings of the Biological Society of Washington 76: 169–172.

Conant R (1963b) Semiaquatic snakes of the genus *Thamnophis* from the isolated drainage system of the Río Nazas and adjacent areas of Mexico. Copeia 1963(3): 473–499.

Conant R (1965) Miscellaneous notes on toads, lizards, and snakes from Mexico. American Museum Novitates 2205: 138.

Cope ED (1885) A contribution to the herpetology of Mexico. Proceedings of the American Philosophical Society 22: 379–404.

Duellman WE, Zweifel RG (1962) A synopsis of the lizards of the *sexlineatus* group (genus *Cnemidophorus*). Bulletin of the American Museum of Natural History 123: 155–210.

Dugés AD (1869) Catálogo de animales vertebrados observados en la república mexicana. La Naturaleza. Serie 1: 137–145.

Dundee HA, Liner EA (1976) Geographic distribution: *Ambystoma tigrinum velasci*. (Plateau Tiger Salamander.). Herpetological Review 7(4): 178.

Feria-Ortiz M, Manríquez-Morán NL, Nieto-Montes de Oca A (2011) Species limits based on mtDNA and morphological data in the polytypic species *Plestiodon brevirostris* (Squamata: Scincidae). Herpetological Monographs 25(1): 25–51.

Fleet RR, Dixon JR (1971) Geographic variation within the long-tailed group of the glossy snake, *Arizona elegans* Kennicott. Herpetologica 27(3): 295–302.

Flores-Villela OA, Hernández-García E, Nieto-Montes de Oca A (1991) Catálogo de anfibios y reptiles del Museo de Zoología. Serie Catálogos del Museo Nacional de Zoología Alfonso L. Herrera (3):1–222.

Flores-Villela OA, Smith EN, Canseco-Márquez L, Campbell JA (2022) A new species of blindsnake from Jalisco, Mexico (Squamata: Leptotyphlopidae). Revista Mexicana de Biodiversidad 93: e933933.

Frost D, Aird S (1978) Geographic distribution: *Rhinocheilus lecontei tessellatus* (Texas Long-nosed Snake). Herpetological Review 9(2): 62.

Gámez-Gallegos MR, Solís-De Ávila LI, Sigala-Valdéz HA, Soto-Bermúdez U, Ponce-Campos P (2024) First record of *Micruroides euryxanthus neglectus* (Serpentes: Elapidae) for the state of Zacatecas. Revista Latinoamericana de Herpetología 7(3): 137–140.

García-Balderas CM, Quintero-Diaz GE (2012) Geographic distribution: *Geophis dugesii* (Dugés' earth snake). Herpetological Review 43(4): 621.

Gloyd HK (1936) A mexican subspecies of *Crotalus molossus* Baird and Girard. Occasional Papers of the Museum of Zoology, University of Michigan (325): 1–5.

Gloyd HK (1940) The rattlesnakes, genera *Sistrurus* and *Crotalus*. Chicago Academy of Science, Special Publication 4, 266.

Grummer JA, Bryson RW Jr (2014) A new species of bunchgrass lizard (Squamata: Phrynosomatidae) from the southern sky islands of the Sierra Madre Occidental, Mexico. Zootaxa 3790(3): 439–450. <http://dx.doi.org/10.11646/zootaxa.3790.3.3>

Grünwald CI, Reyes-Velasco J, Franz-Chávez H, Morales-Flores KI, Ahumada-Carrillo IT, Rodríguez CM, Jones JM (2021) Two new species of *Eleutherodactylus* (Anura: Eleutherodactylidae) from Southern Mexico, with comments on the taxonomy of related species and their advertisement calls. Amphibian & Reptile Conservation 1–35.

Iverson JB (1985) *Kinosternon hirtipes* (Wagler) Mexican rough-footed mud turtle. Catalogue of American Amphibians and Reptiles 361: 1–4.

Iverson JB, Young CA, Berry JF (1998) *Kinosternon integrum* LeConte. Catalogue of the American Amphibians and Reptiles 652: 1–6.

Johnson JD (1977) The taxonomy and distribution of the neotropical whipsnake *Masticophis mentovarius* (Reptilia, Serpentes, Colubridae). Journal of Herpetology 11(3): 287–309.

Keiser ED Jr (1969) An unusual specimen of the neotropical vine snake, *Oxybelis aeneus* (Wagler) from Zacatecas, Mexico. British Journal of Herpetology 4(5): 116–117.

Kellogg R (1932) Mexican tailless amphibians in the United Sates National Museum. Smithsonian Institution. United States National Museum. Bulletin 160: 27–31.

Krupa JJ (1990) *Bufo cognatus* Say Great Plains Toad. Catalogue of American Amphibians and Reptiles. Society for the Study of Amphibians and Reptiles 457.1–457.8.

Liner EA, Dundee HA (1977) Geographic distribution: *Lampropeltis mexicana mexicana* (Mexican Kingsnake). Herpetological Review 8: 85.

McCoy CJ Jr (1961) Additional records of *Ficimia cana* from Mexico and Texas. Herpetologica 17(3): 215.

McCoy CJ (1964) Notes on Snakes from Northern Mexico. The Southwestern Naturalist 9(1): 46. <http://dx.doi.org/10.2307/3669106>

McCranie JR (1977) First record of *Tantilla bocourti* (Reptilia: Colubridae) from Zacatecas, Mexico. The Southwestern Naturalist 22(2): 275.

Morafka DJ (1977) A biogeographical analysis of the Chihuahua Desert through its herpetofauna. W Junk BV, Publisher, The Hague. vii+313.

Myers CW (1974) The systematics of *Rhadinaea* (Colubridae), a genus of New World snakes. Bulletin of the American Museum of Natural History 153(1): 1–262.

Parker RB (1960) The status of the Mexican lizard, *Eumeces lynxe belli*. Copeia 1960(4): 284–286.

Price AH (1990) *Phrynosoma cornutum* (Harlan): Texas Horned Lizard. Catalogue of American Amphibians and Reptiles 469: 1–7.

Quintero-Díaz GE, Pérez-Ramos E, Carbajal-Márquez RA (2015) *Lithobates magnaocularis* Frost & Bagnara, 1974, the Northwest Mexico Leopard Frog (Anura: Ranidae): new state records for Aguascalientes and Zacatecas, Mexico. Check List 11(6): 1804. <https://doi.org/10.15560/11.6.1804>

Rodríguez-Maturino A, Viggers-Carrasco MG, Villa-López MM, Valdez-Lares R, Pulido-Marrufo LR, Soto-Olvera BY, Gómez-Rivera KS, Fernández-García JA, Ruiz-Díaz H (2018) Reptiles del Parque Nacional Sierra de Órganos, Zacatecas. Áreas Naturales Protegidas Scripta 4(1): 1–23. <https://doi.org/10.18242/anpscripta.2018.04.04.01.0001>

Rosales-Martínez CS, Díaz-Valdivia CE, Cupul-Magaña FG (2022) Nota de Distribución: *Terrapene nelsoni* (Emydidae). Revista Latinoamericana de Herpetología 5(4):n 120–122.

Rossman DA (1971) Systematics of the Neotropical populations of *Thamnophis marcianus* (Serpentes: Colubridae). Occasional Papers of the Museum of Zoology, Louisiana State University 41: 13.

Schmidt KP (1922) A review of the North American genus of lizards *Holbrookia*. Bulletin of the American Museum of Natural History 46(12): 709–725.

Sigala-Rodríguez JJ, Quintero-Díaz GE, Ahumada-Carrillo IT, Carbajal-Márquez RA, Enríquez-Enríquez ED, Vacio-de la Torre MR (2020b) Reptiles. In: La Biodiversidad en Zacatecas. Estudio de Estado. CONABIO, México, 235–240.

Smith HM (1935) Miscellaneous notes on Mexican lizards. University of Kansas Science Bulletin 22: 119–156.

Smith HM (1938) Remarks on the status of the subspecies of *Sceloporus undulatus*, with descriptions of new species and subspecies of the *Undulatus* group. Occasional Papers of the Museum of Zoology, University of Michigan 387: 1–17.

Smith HM (1939) The Mexican and Central American lizards of the genus *Sceloporus*. Field Museum of Natural History, Zoological Series 26: 1–397.

Smith HM (1942) Mexican Herpetological Miscellany. Proceedings of the United States National Museum. Smithsonian Institution 92(3153): 349–395.

Smith HM, Taylor EH (1945) An annotated checklist and key to the snakes of Mexico. Smithsonian Institution United States National Museum. Bulletin 199: 94–104.

Smith HM, Taylor EH (1948) An Annotated Checklist and key to the amphibia of Mexico. Smithsonian Institution United States National Museum. Bulletin 187: 77–84.

Smith HM, Taylor EH (1950) An Annotated Checklist and key to the reptiles of Mexico exclusive of the snakes. Smithsonian Institution United States National Museum. Bulletin 199: 94–104.

Smith NM, Tanner WW (1974) A taxonomy study of the western collared lizards, *Crotaphytus collaris* and *Crotaphytus insularis*. Brigham Young University. Science Bulletin 19(4): 1–29.

Taylor EH (1936) Notes on the Herpetological Fauna of the Mexican State of Sinaloa. The University of Kansas. Science Bulletin 24(20): 505–537.

Taylor EH, Smith HM (1942) The snake genera *Conopsis* and *Toluca*. Science Bulletin. The University of Kansas 28(2): 325–363.

Villa RA, Carrillo-Reyes P, Ávila-Villegas H (2011) Geographic distribution: *Mastigodryas cliftoni* (Clifton´s Lizard Eater). Herpetological Review 42(4): 573.

Villalobos-Juárez I (2023). Primer registro de la rana de madriguera *Smilisca dentata* (Smith, 1957) para el estado de Zacatecas, México. Revista Latinoamericana de Herpetología, 6(2): e637 (10 – 13). <https://doi.org/10.22201/fc.25942158e.2023.2.637>

Villalobos-Juárez I, García-Padilla E (2023) Nuevos registros de anfibios y reptiles para el centro de México. Revista Latinoamericana de Herpetología, 6(1): 92–94. <https://doi.org/10.22201/fc.25942158e.2023.01.638>

Villalobos-Juárez I, García-Padilla E, Heredia-López I, Lazcano D (2025) First Record of the Ornate Black-tailed Rattlesnake (*Crotalus ornatus*) in the State of Zacatecas, Mexico. Bulletin of the Chicago Herpetological Society 60(6): 79-80.

Webb RG (1970) *Gerrhonotus kingii* (Gray) Sonoran alligator lizard. Catalogue of American Amphibians and Reptiles 97: 1–4.

Webb RG (1976) A review of the garter snake *Thamnophis elegans* in Mexico. Los Angeles County the Natural History Museum Bulletin 284: 1–13.

Webb RG (1980) *Thamnophis cyrtopsis*. Catalogue of American Amphibians and Reptiles 245: 1–4.

Webb RG (1982) Geographic distribution: Distributional records for Mexican reptiles. Herpetological Review 13(4): 132.

Williams KL (1968) A new subspecies of the teiid lizard *Cnemidophorus inornatus* from México. Journal of Herpetology 1(1–4): 21–24.

Wilson LD, McCranie JR (1979) Notes on the Herpetofauna of Two Mountain Ranges in México (Sierra Fría, Aguascalientes, and Sierra Morones, Zacatecas). Journal of Herpetology 13(3): 271–278.

Zweifel RG (1954) Notes on the distribution of some reptiles in western Mexico. Herpetologica 10:145–149.

Zweifel RG (1959) Variation in and distribution of lizards of western Mexico related to *Cnemidophorus sacki*. Bulletin of the American Museum of Natural History 117(2): 57–116.
