# Supplementary material for: Annotated checklist of the amphibians and reptiles of Zacatecas, Mexico
Source: Zookeys. 2026 Jun 2;1281:21–48. doi: 10.3897/zookeys.1281.174112 (PMC13250616; doi:10.3897/zookeys.1281.174112)
Supplement: Supplementary material 3 — List of species with complete metadata that represent new state and municipality records for Zacatecas, Mexico. * = new state record [file zookeys-1281-021_article-174112__-s003.docx]

**Appendix 3** List of species with complete metadata that represent new state and municipality records for Zacatecas, Mexico. * = new state record.

**Abbreviation notes (for collectors):** **AEL** = Alondra Encarnación-Luévano; **AIHG** = Aranxa Isabel Hernández-González; **BYV:** Byanca Yazmin Velázquez; **CCS** = Carlos Carrillo-Sánchez; **CEEE** = Citlalli Edith Esparza-Estrada; **CFSM =** Cristian Franco-Servín de la Mora; **DAR =** David Andrade-Rubalcava; **DCSM =** Diego César Sánchez-Marrufo; **DJM =** David J. Morafka; **DMP** = David Morales-Pánuco; **DSCL =** Diana Stephanie Carrillo-Lara; **EAMS** = Elizabeth Aurelia Martínez-Salazar**; ECA** = Eric Centenero-Alcalá; **EDEE** = Enrique David Enríquez-Enríquez; **EEGM** = Emmanuel Eduardo González-Medina; **EJRP** = Emmeth Josafath Rodríguez-Pérez; **EMR** = Ernestina Meza-Rangel; **EOG** = Edgar Octavio González; **EP** = Elizabeth Painter; **ESM** = Eric Sigala-Meza; **GEQD** = Gustavo Ernesto Quintero-Díaz; **GGJ =** Gerardo Guerrero-Jiménez; **GMV =** Guillermo Martínez-de la Vega; **ITAC =** Iván Trinidad Ahumada-Carrillo**; IVJ** = Iván Villalobos-Juárez; **JABA** = Jorge Alberto Bañuelos-Alamillo; **JCAM** = José Carlos Arenas-Monroy; **JCF** = Jaime Carrillo-Fernandez; **JEDP** = José Eduardo Díaz-Placencia; **JEVV** = Jesús Enrique Valencia-Villegas **JFMM:** Juan Felipe Martínez-Montoya; **JJAR** = Juan José Ayala-Rodríguez; **JJDS** = Joanne Jessica Delgado-Saucedo; **JJSR** = Jose Jesús Sigala-Rodríguez; **JLLG:** Jesús Lenin Lara-Galván; **JLR** = Jesús Lara-Rayos; **JMTG** = José Manuel Trujillo-González; **JR** = Jared Rangel; **LAH** = Lucía Ávila-Herrera; **LIAL** = Luis Ignacio Almaráz-Llamas**; LM =** Larry Michel; **MAL =** Marco Antonio López; **MGAA** = María Guadalupe Ávila-Ávila; **MLDT** = Mónica Lizeth Díaz-Teniente; **MN** = Manuel Nevárez; **MNe** = Mike Newlin; **MW** = M. White; **NYGA** = Nallely Yosajandi Gamboa-Arteaga; **OMG** = Oscar Medina-García**; OVR** = Oscar Vázquez-Reyes; **PGM** = Patricia García-Macías**; RACM** = Rubén Alonso Carbajal-Márquez; **RARG** = Ramsés Alejandro Rosales-García; **RC** = Rafael Cervantes; **RED** = Rubén Edgardo-Diaz; **RGW** = Robert G. Webb; **RIMV =** Rebeca Isela Morales-Valerio; **RN =** Rodolfo Núñez; **RO** = Ramón Orozco; **RRG** = Rogelio Rosales-García; **RRM** = Renato Rivera-Menchaca; **RVC** = Roberto Vizcaya-Caballero; **SGMG =** Samira Gretel Mata-García; **SHAR** = Salvador Héctor Acevedo-Rodríguez; **SSM** = Sofía Sigala-Meza.

| **Species** | **Municipality** | **Locality** | **Latitude** | **Longitude** | **Elevation** | **Date** | **Catalog number** | **Collectors** |
| --- | --- | --- | --- | --- | --- | --- | --- | --- |
| *Ambystoma rosaceum* | Monte Escobedo | 8.5 km W of Monte Escobedo | 22.318918 | -103.641749 | 2456 | 2011/03/00 | CZUAA-ANF 234-235 | CCS, MGAA and JJSR |
| *Ambystoma velasci* | Mazapil | San Tiburcio | 24.2109 | -101.5806 | 2016 | 12/07/2012 | CNAR-RF-939 | ECA |
|  | Mazapil | San Tiburcio | 24.1762 | -101.5149 | 1944 | 10/08/2012 | CNAR-RF-941 | ECA |
|  | Valparaíso | 25.2 km E Valparaíso | 22.8142 | -103.3278 | 2187 | 20/09/2018 | UTADC 9905 | JABA |
|  | Villa de Cos | 14.4 km SE Majoma | 23.8078 | -101.5837 | 2128 | 13/06/2015 | CZUAA-ANF-330 | JJSR |
| *Anaxyrus cognatus* | Mazapil | Peñasquito | 24.6645 | -101.7142 | 1907 | 08/09/2015 | CNAR-RF-944 | ECA |
|  | Pinos | Pinos Town | 22.3201194 | -101.5387194 | 2637 | 20/06/2018 | UTADC 10005 | JLLG and JFMM |
| *Anaxyrus compactilis* | Atolinga | Crop field near the road before reaching Atolinga | 21.8116 | -103.3973 | 2138 | 05/05/2010 | CZUAA-ANF-258 | JLLG and JJSR |
|  | Genaro Codina | El Palmarito | 22.4751778 | -102.448980 | 2134 | 12/09/2013 | UTADC 10006 | JLLG |
|  | Monte Escobedo | 8.3 km W of Monte Escobedo | 22.318059 | -103.640800 | 2457 | 23/09/2011 | CZUAA-ANF-246 | MGAA and JJSR |
|  | Nochistlán de Mejía | North of "La Estancia" | 21.4439 | -102.7526 | 1974 | 20/09/2011 | UTADC 9927 | JJSR |
| *Anaxyrus punctatus* | Juchipila | 7.6 km SW of Juchipila | 21.382 | -103.1823 | 1632 | 10/08/2014 | UTADC 9928 | EMR, ESM and JJSR |
|  | Monte Escobedo | 8.2 km W of Monte Escobedo | 22.319436 | -103.639364 | 2441 | 23/09/2011 | CZUAA-ANF-366 Y 367 | MGAA and JJSR |
|  | Villa García | 1.5 km to NE of Villa García Town | 22.1678778 | -101.9415333 | 2133 | 18/06/2017 | UTADC 10007 | JLLG and JFMM |
| *Incilius occidentalis* | Juchipila | Sierra Morones | 21.402 | -103.1912 | 2017 | 13/09/2009 | CZUAA-ANF-268 | JJSR |
|  | Monte Escobedo | 3.7 km S of Monte Escobedo | 22.2706 | -103.5498 | 2070 | 15/10/2011 | CZUAA-ANF-336 | CCS, MGAA and JJSR |
|  | Sain Alto | El Cazadero Dam | 23.688532 | -103.109887 | 1927 | 21/10/2011 | CZUAA-ANF-260 | JJSR |
|  | Tlaltenango | 7.9 km airline SW Gral. Joaquin Amaro | 21.8954 | -103.1554 |  | 14/08/2010 | SDNHM_HerpPC 05449 | RACM |
|  | Trinidad de la Cadena García | Las Higueras | 21.1873 | -103.5308 | 1257 | 30/10/2010 | CZUAA-ANF-368 | JCAM and JJSR |
|  | Villanueva | Tayahua | 22.092552 | -102.855879 | 1761 | 07/10/2011 | CZUAA-ANF-244 | JJSR |
| *Rhinella horribilis* | Trinidad García de la Cadena | Río Patitos, road from García de la Cadena to Las Higueras | 21.1826 | -103.5322 | 1312 | 31/10/2010 | CZUAA-ANF-228 | JJSR |
|  | Valparaíso | San Juan Capistrano | 22.6207028 | -104.0948194 | 1149 | 11/08/2018 | UTADC 10019 | JLLG, JABA, MLDT and EJRP |
|  | Valparaíso | San Juan Capistrano | 22.6313333 | -104.1129722 | 1210 | 12/08/2018 | UTADC 10020 | JLLG, JABA, MLDT and EJRP |
| *Craugastor augusti* | Huanusco | Cerro Chino | 21.7036 | -102.7867 | 2124 | 26/09/2014 | CZUAA-ANF-231 | PGM and JJSR |
|  | Nochistlán de Mejía | 1.6 km SE Palo Herrado | 21.374 | -102.9485 | 2272 | 04/08/2022 | CZUAA-ANF-375 | GEQD |
| *Craugastor occidentalis* | Valparaíso | 14.8 km W of Tenzompa | 22.3739 | -104.0703 | 1104 | 03/09/2018 | UTADC 9897 | JABA |
| *Eleutherodactylus jamesdixoni* | Jerez | Stream parallel to the road that goes to Palmas Altas, 1.68 km NW of Parral de las Huertas | 22.7335 | -103.1015 | 2273 | 03/07/2014 | CZUAA-ANF-286 | RARG |
|  | Jiménez del Teúl | Talus 5.5 km W of Mimbres | 22.8664 | -103.5828 | 2272 | 10/09/2010 | CZUAA-ANF-308 | LIAL, MGAA, R and JJSR |
|  | Tlaltenango de Sánchez Román | 1.17 km N El Puertecito | 21.6653 | -103.1559 | 2478 | 23/08/2017 | CZUAA-ANF-355 | SSM and JJSR |
| *Eleutherodactylus guttilatus** | Pinos | Saldaña | 22.384457 | -101.386169 | 2324 | 22/09/2014 | CZUAA-ANF-309 | JJSR |
| *Dryophytes arenicolor* | Cuauhtémoc | San Pedro | 22.439 | -102.3884 | 2129 | 01/10/2011 | CZUAA-ANF-247 | EAMS and JJSR |
|  | Huanusco | 2.3 km S San Rafael | 21.6826 | -102.7769 | 2309 | 17/06/2011 | UTADC 9929 | JJSR |
|  | Monte Escobedo | San Isidro | 22.3019056 | -103.5814 | 2324 | 26/07/2011 | UTADC 10008 | JLLG and EJRP |
|  | Nochistlán de Mejía | Rancho El Sombreretillo | 21.6007972 | -102.785 | 2191 | 15/04/2017 | UTADC 10014 | JLLG and EP |
|  | Pinos | El Chiquihuitillo, La Lobeña | 22.2995056 | -101.70900833 | 2220 | 18/09/2013 | UTADC 10010 | JLR and JLLG |
|  | Sombrerete | 1.3 km SE San Martin | 23.6619 | -103.7415 | 2625 | 04/09/2020 | UTADC 9922 | ITAC |
|  | Susticacán | Susticacán Town | 22.6089 | -103.0975027 | 2033 | 29/09/2014 | UTADC 10012 | JLLG |
|  | Zacatecas | Cerro de La Virgen | 22.7436917 | -102.56310555 | 2695 | 26/09/2014 | UTADC 10011 | JLLG, MLDT, EJRP and DMP |
| *Dryophytes eximius* | Cuauhtémoc | Presa San Pedro | 22.443 | -102.3814 | 2051 | 29/10/2011 | UTADC 9930 | JJSR |
|  | Huanusco | Las Monas | 21.7041 | -102.7795 | 2160 | 22/07/2013 | SDNH_HerpPC 05446 | RACM |
|  | Nochistlán de Mejía | 11 km N Nochistlán de Mejía | 21.4595 | -102.8348 | 2340 | 23/09/2013 | UTADC 9931 | DSCL and JJSR |
| *Hypopachus variolosus* | Apulco | 2.3 km NE Tenayuca | 21.5182 | -102.6422 | 1941 | 03/07/2015 | CZUAA-ANF-267 | EMR and JJSR |
|  | Genaro Codina | Ojo de Agua, Genaro Codina | 22.4922833 | -102.451055 | 2234 | 21/06/2016 | UTADC 10017 | JLLG |
|  | Momax | 1.73 km NW to Momax Town | 21.9346139 | -103.3242413 | 1635 | 10/10/2021 | UTADC 10018 | JLLG |
|  | Valparaíso | 3.3 km S of San Juan Capistrano | 22.6128 | -104.0897 | 1145 | 29/08/2015 | UTADC 9898 | JABA |
| *Aquarana catesbeiana** | Apulco | 2.6 km N Tenayuca | 21.5266 | -102.6524 | 1957 | 03/07/2015 | CZUAA-ANF-243 | EMR and JJSR |
| *Lithobates magnaocularis* | Mezquital del Oro | Los Cardos Dam | 21.2701 | -103.3033 | 1610 | 01/06/2011 | CZUAA-ANF-360 | RVC and JJSR |
|  | Nochistlán de Mejía | 5.3 km S Nochistlán de Mejía | 21.316 | -102.8538 | 1977 | 07/10/2011 | CZUAA-ANF-364 | JJSR |
|  | Trinidad García de la Cadena | 1.7 km W Trinidad García de la Cadena | 21.2121 | -103.4805 | 1723 | 31/10/2010 | CZUAA-ANF-276 | JCAM and JJSR |
| *Lithobates montezumae* | Apulco | 2 km NE Tenayuca | 21.5182 | -102.6482 | 1944 | 03/07/2015 | CZUAA-ANF-265 | JJSR and EMR |
|  | Genaro Codina | San Pedro Dam | 22.472 | -102.4402 | 2096 | 14/06/2012 | CZUAA-ANF-371 | CEEE and JJSR |
|  | Huanusco | 1.9 km SE San Rafael | 21.6839 | -102.7821 | 2320 | 17/06/2011 | CZUAA-ANF-361 | JJDS and JJSR |
|  | Mezquital del Oro | Los Cardos Dam | 21.2701 | -103.3033 | 1613 | 01/06/2011 | CZUAA-ANF-359 | JJSR and RVC |
|  | Zacatecas | Infante Dam | 22.7795 | -102.5442 | 2403 | 31/10/2011 | CZUAA-ANF-369 | JJSR |
| *Scaphiopus couchii* | El Salvador | 6.12 km NE from El Salvador Town, near tripoint Coahuila, Zacatecas, Nuevo León | 24.5416833 | -100.8097805 | 1786 | 30/08/2016 | UTADC 10021 | JLLG |
|  | Mazapil | 2.5 km E General Felipe Ángeles | 24.632 | -101.9494 | 1712 | 21/06/2015 | CZUAA-ANF 329 | JJSR |
| *Spea multiplicata* | El Salvador | 6 km NE from El Salvador Town, near tripoint Coahuila, Zacatecas, Nuevo León | 24.5634389 | -100.90057222 | 1750 | 26/08/2017 | UTADC 10023 | CEEE, JLLG and JJSR |
|  | General Francisco R. Murguía | Calabacitas stream at 1.3 km NE La Estanzuela | 23.9473 | -103.1208 | 1948 | 19/08/2011 | CZUAA-ANF-372 | JJSR |
|  | Jalpa | 3.4 km SW Jalpa | 21.6174 | -103.0068 | 1421 | 28/07/2011 | CZUAA-ANF-374 | JJSR and RVC |
|  | Jerez | 0.7 Km SE Santa Fe | 22.5348 | -103.0365 | 1939 | 16/09/2016 | CZUAA-ANF-283 | RARG and RRG |
|  | Monte Escobedo | 16 km NE Laguna Grande | 22.5453 | -103.3969 | 2368 | 14/08/2010 | CZUAA-ANF-257 | JCF, MGAA, LIAL and JJSR |
|  | Sombrerete | 1.2 km NE San Martin | 23.6739 | -103.7367 | 2550 | 14/09/2020 | UTADC 9923 | ITAC |
|  | Zacatecas | Cerro de La Virgen | 22.7445694 | -102.5580277 | 2650 | 09/09/2015 | UTADC 10022 | JLLG |
| *Kinosternon hirtipes* | Valparaíso | 2.8 km E of Valparaíso | 22.7768 | -103.5411 | 1892 | 08/10/2011 | UTADC 9907 | JABA |
| *Kinosternon integrum* | El Plateado Joaquín Amaro | 4.1 km SE El Plateado | 21.9175 | -103.0564 | 2454 | 29/09/2011 | UTADC 9933 | EMR and JJSR |
|  | Fresnillo | 7.6 km SE Fresnillo | 23.1318 | -102.8108 | 2155 | 21/06/2015 | UTADC 9934 | EMR, SSM and JJSR |
|  | Nochistlán de Mejía | 2.6 km N Tlachichila | 21.5818 | -102.7804 | 2142 | 27/08/2011 | UTADC 9932 | JJSR |
|  | Valparaíso | 2.8 km E of Valparaíso | 22.7768 | -103.5411 | 1892 | 08/10/2011 | UTADC 9906 | JABA |
| *Gopherus berlandieri** | Melchor Ocampo | 2.5 km S El Jaguey | 24.7099 | -101.6678 | 2115 | 10/07/2017 | CNAR-RF-954 | RC and ECA |
| *Barisia ciliaris* | Genaro Codina | Sierra Fría in Genaro Codina, close to the border with Aguascalientes | 22.29224 | -102.58382 | 2804 | 24/10/2014 | UTADC 10029 | JJSR, CEEE, PGM, JLLG and RARG |
|  | Jerez | Sierra de Cardos, 0.6 km W El Portillo. | 22.7078 | -103.1363 | 2542 | 22/08/2014 | CZUAA-REP-630 | RARG and JJSR |
|  | Monte Escobedo | 8.2 km W Monte Escobedo | 22.318993 | -103.641457 | 2451 | 23/09/2011 | CZUAA-REP-1006 | MGAA, NYGA and JJSR |
|  | Sombrerete | 1.3 km SE San Martin | 23.6598 | -103.7426 | 2630 | 08/09/2020 | UTADC 9924 | ITAC |
|  | Tlaltenango | 7.9 km airline SW Gral. Joaquín Amaro | 21.8954 | -103.1554 | 2598 | 14/08/2010 | SDNH_HerpPC 05439 | RACM |
| *Elgaria kingii* | Juchipila | Stream in Rancho El Piñón | 21.3483 | -103.242 | 2091 | 18/10/2014 | CZUAA-REP-540 | JJSR |
|  | Valparaíso | 1.2 km N Santa Cruz de Tepetates | 22.5327 | -104.3209 | 2599 | 13/08/2020 | CZUAA-REP-892 | JABA, JJAR and JJSR |
| *Gerrhonotus infernalis* | Concepción del Oro | 7.2 km SW Las Huertas | 24.3634 | -101.16 | 2206 | 01/09/2011 | CZUAA-REP-456 | RRM and JJSR |
|  | Mazapil | 13.6 km NW of Mazapil | 24.674 | -101.681 | 2258 | 19/10/2020 | UTADC 9926 | ITAC |
| *Crotaphytus collaris* | El Salvador | 6 km NE from El Salvador Town, near tripoint Coahuila, Zacatecas, Nuevo León | 24.5661917 | -100.9006944 | 1752 | 26/08/2017 | UTADC 10031 | JLLG, CEEE and JJSR |
| *Gambelia wislizenii** | Mazapil | Novillos | 24.7147917 | -101.5539194 | 2044 | 23/07/2017 | UTADC 10032 | JLLG |
|  | Melchor Ocampo | Matamoros | 24.9034 | -102.0252 | 1523 | 20/06/2015 | CNAR-RF-931 | ECA |
| *Anolis nebulosus* | Chalchihuites | 9.2 km E of Ojo de Agua | 23.3723 | -103.7643 | 2405 | 27/01/2023 | UTADC 9912 | ITAC |
|  | Huanusco | 2.3 km SE San Rafael | 21.681789 | -102.777018 | 2259 | 17/06/2011 | UTADC 9935 | JJSR |
|  | Jiménez del Teúl | 8.7 km SW Jiménez del Teúl | 23.2124 | -103.872 | 2164 | 26/07/2012 | CZUAA-REP-1007 | OVR and JJSR |
|  | Nochistlán de Mejía | Rancho el Sombreretillo | 21.60362 | -102.78457 | 2287 | 10/10/2016 | UTADC 10024 | JLLG and EP |
| *Coleonyx brevis* | Mazapil | Terminal de Providencia | 24.6809 | -101.4688 | 2046 | 25/08/2015 | CNAR-RF-937 | ECA |
|  | Mazapil | Terminal de Providencia | 24.6788 | -101.4684 | 2072 | 25/08/2015 | CNAR-RF-938 | ECA |
|  | Melchor Ocampo | 2.2 km N San Juan de los Cedros | 24.6980 | -101.7756 | 1780 | 19/10/2020 | UTADC 9914 | ITAC |
| *Hemidactylus turcicus** | Chalchihuites | Chalchihuites | 23.4669 | -103.8826 | 2302 | 29/09/2020 | https://www.naturalista.mx/observations/61277459 | RED |
|  | Concepción del Oro | Concepción del Oro | 24.6143 | -101.4172 | 2070 | 26/09/2018 | https://www.naturalista.mx/observations/17122257 | RED |
|  | General Enrique Estrada | General Félix U. Gómez | 23.0485 | -102.7729 | 2156 | 01/04/2022 | https://www.naturalista.mx/observations/110084509 | JR |
|  | Mazapil | San Tiburcio | 24.1466 | -101.4856 | 1891 | 07/10/2020 | https://www.naturalista.mx/observations/61998880 | MN |
| *Ctenosaura pectinata* | Apozol | 0.8 km airline E Los Llamas | 21.5030 | -103.0823 | 1298 | 23/03/2009 | SDNH_HerpPC 05444 | RACM |
|  | Monte Escobedo | 21 km airline S Monte Escobedo | 22.1128 | -103.5797 | 1252 | 11/03/2007 | SDNH_HerpPC 05445 | GEQD, AEL, RACM and GMV |
|  | Trinidad García de la Cadena | Las Higueras | 21.1836 | -103.535 | 1238 | 31/10/2010 | CZUAA-REP-1008 | JCAM and JJSR |
|  | Valparaíso | Los Medina, 4.6 km N Canelos | 23.0001 | -104.0798 | 1313 | 09/10/2010 | CZUAA-REP-419 | OMG, LAH and JJSR |
| *Holbrookia approximans* | Pánfilo Natera | San José el Saladillo | 22.7064 | -102.0280 | 2035 | 21/10/2017 | UTADC 9915 | ITAC |
|  | Villa González Ortega | 5.22 JM N from Villa González Ortega Town | 22.537 | -101.8706583 | 2164 | 07/07/2017 | UTADC 10034 | JLLG |
| *Phrynosoma cornutum* | Mazapil | 14.3 km SW El Peñasquito | 24.6478 | -101.8868 | 1661 | 05/07/2010 | CZUAA-REP-625 | JJSR |
| *Phrynosoma modestum* | Concepción del Oro | 4.1 km SW Las Huertas | 24.3801 | -101.1371 | 2008 | 01/09/2011 | UTADC 9936 | RRM, DSCL, AIHG, NYGA and JJSR |
|  | Villa González Ortega | Close to Villa González Ortega Town | 22.5284083 | -101.9160777 | 2171 | 01/04/2017 | UTADC 10040 | JLLG and EP |
| *Phrynosoma orbiculare* | Monte Escobedo | 3.7 km SE Monte Escobedo | 22.2706 | -103.5498 | 2070 | 15/10/2011 | CZUAA-REP-514 | CCS and MGAA |
|  | Noria de Ángeles | 3.16 km NW from Noria de Ángeles | 22.4611611 | -101.929275 | 2164 | 01/08/2018 | UTADC 10041 | JLLG |
|  | Susticacán | Susticacán Town | 22.6064167 | -103.09308888 | 2026 | 11/07/2017 | UTADC 10042 | JLLG |
| *Phrynosoma ornatissimum* | Jiménez del Teúl | Luis Moya de Arriba | 23.1893 | -103.6954 | 2187 | 21/11/2011 | CZUAA-REP-618 | OVR |
| *Sceloporus albiventris** | Chalchihuites | 10.7 km E Ojo de Agua | 23.3644 | -103.7484 | 2375 | 20/03/2020 | UTADC 9911 | ITAC |
| *Sceloporus aurantius* | Monte Escobedo | 8.5 k m W Monte Escobedo | 22.318192 | -103.642944 | 2461 | 23/09/2011 | UTADC 9937 | MGAA and JJSR |
|  | Teúl de González Ortega | 7.1 km S Teúl de González Ortega | 21.4051 | -103.4899 | 2026 | 25/03/2012 | UTADC 9910 | ITAC |
| *Sceloporus bimaculosus** | Villa de Cos | 73 mi N of jct. Mex. 54/45 on Mex. 54 (jct. 19 km NW of Zac, Zac.) | 23.7000 | -101.8900 | 2148 | 21/07/1971 | CMNH 59651 | DJM |
| *Sceloporus brownorum* | Tepetongo | Near to El Salitre and La Cuadrilla | 22.47855 | -103.1676472 | 1968 | 11/07/2017 | UTADC 10046 | JLLG |
|  | Valparaíso | 0.7 km N Santa Cruz de Tepetates | 22.5327 | -104.3209 | 2632 | 14/08/2020 | CZUAA-REP-890 | JJSR |
| *Sceloporus cautus* | Mazapil | 6.4 km El Peñasquito | 24.6254 | -101.7934 | 1700 | 27/08/2019 | CNAR-RF-952 | ECA |
|  | Mazapil | San Tiburcio | 24.1578 | -101.5039 | 1910 | 23/06/2012 | CNAR-RF-935 | ECA |
|  | Pánfilo Natera | San José El Saladillo | 22.687861 | -102.0337 | 2023 | 29/11/2019 | UTADC 9909 | ITAC |
| *Sceloporus clarkii* | Tepechitlán | 3.3 km SW Tepechitlán | 21.6469 | -103.3464 | 1748 | 19/11/2011 | CZUAA-REP-641 | BYV and JJSR |
| *Sceloporus dugesii* | Monte Escobedo | 8 km NW to Monte Escobedo | 22.320825 | -103.639101 | 2445 | 14/09/2011 | CZUAA-REP-672 y 673 | JJSR, MGAA, JLLG and MLDT |
|  | Tepetongo | Presa la cuadrilla | 22.4943306 | -103.1619361 | 1975 | 11/06/2012 | UTADC 10047 | JLLG |
| *Sceloporus horridus* | Jerez | 1.79 km N Parral de las Huertas | 22.738 | -103.0899 | 2351 | 27/09/2014 | CZUAA-REP-629 | RARG, RRG and CFSM |
|  | Juchipila | Stream at Rancho el Piñon | 21.3486 | -103.2433 | 2067 | 18/10/2014 | CZUAA-REP-674 | JJSR |
|  | Momax | 1.5 km W Momax | 21.9198 | -103.328 | 1671 | 18/08/2011 | CZUAA-REP-1011 | LIAL, NYGA, LAH, RRM and JJSR |
|  | Tabasco | 1.4 km NW Huiscolco | 21.9015 | -102.9559 | 1608 | 12/08/2012 | CZUAA-REP-1010 | EMR and JJSR |
| *Sceloporus huichol* | Monte Escobedo | 8.1 km W Monte Escobedo | 22.3234 | -103.6452 | 2404 | 15/10/2011 | CZUAA-REP-1012 | MGAA and CCS |
|  | Valparaíso | Valparaíso. | 22.7704 | -103.5698 | 1898 | 22/10/2011 | CZUAA-REP-1018 | JJSR |
| *Sceloporus olivaceus** | Mazapil | 5 km W San Tiburcio | 24.1426 | -101.5393 | 1937 | 04/07/2012 | UTADC 9964 | ECA |
| *Sceloporus ornatus** | Mazapil | East slope Pico de Teyra | 24.5578 | -102.1735 | 2694 | 18/09/2010 | CZUAA-REP-847-849 | LIAL, SHAR and JJSR |
| *Sceloporus parvus* | Mazapil | 5.8 km E Mazapil | 24.6405 | -101.4973 | 2743 | 17/09/2010 | CZUAA-UAA-REP 521 | SHAR, LIAL and JJSR |
| *Sceloporus cf. scalaris** | Concepción del Oro | 80 mi S Saltillo on HWY 57 | 24.3473 | -101.2086 | 2102 | 25/12/1972 | ASNHC 10372 | MW, MNe and LM |
|  | Concepción del Oro | 7 km SW Las Huertas | 24.363 | -101.1584 | 2180 | 02/09/2011 | CZUAA-UAA-REP 617 | NYGA and JJSR |
| *Sceloporus shannonorum* | Jerez | El Portillo, carretera Jerez a Sierra de Cardos | 22.711781 | -103.12007 | 2607 | 23/08/2014 | CZUAA-UAA-REP-587 | RARG |
|  | Monte Escobedo | 3.6 km S Monte Escobedo | 22.2706 | -103.5498 | 2125 | 15/10/2011 | UTADC 9938 | CCS, MGAA and JJSR |
| *Sceloporus utiformis* | Trinidad García de la Cadena | 0.3 km N Las Higueras | 21.185 | -103.5339 | 1296 | 31/10/2010 | CZUAA-UAA-REP-450 | JCAM and JJSR |
| *Urosaurus bicarinatus* | Jiménez del Teúl | 9.3 km SW Jiménez del Teúl | 23.2124 | -103.878 | 2176 | 27/06/2010 | CZUAA-REP-529 y CZUAA-REP-535 | OVR and JJSR |
|  | Jiménez del Teúl | Arroyo del Tecolote, Rancho la Cuchilla, 4.3 km SW Jiménez del Teúl | 23.2272 | -103.8272 | 1953 | 12/05/2012 | CZUAA-REP-1020 | OVR and JJSR |
|  | Valparaíso | 3.4 km SW Romerillo del Sur | 22.6023 | -103.6808 | 2008 | 21/08/2010 | CZUAA-REP-530 | LIAL, NYGA, SHAR and JJSR |
|  | Valparaíso | Talus 5.5 km W Mimbres, | 22.8664 | -103.5828 | 2269 | 11/09/2010 | CZUAA-REP-632 | LIAL, MGAA, SHAR and JJSR |
|  | Valparaíso | Rancho Los Medina, 4.6 km N Canelos | 23.0001 | -104.0798 | 1311 | 09/10/2010 | CZUAA-REP-1021 | LAH, OMG and JJSR |
| *Plestiodon bilineatus* | Jerez | El Portillo Cardos, Sierra de Cardos | 22.7109 | -103.1314 | 2588 | 23/08/2014 | CZUAA-REP-627 | RARG, JJAR, JEDP, CEEE, PGM and JJSR |
| *Plestiodon lynxe* | Huanusco | Cerro Chino, 2.6 km NE San Rafael | 21.7123 | -102.7722 | 2307 | 26/09/2014 | UTADC 9939 | JJSR and PGM |
|  | Nochistlán de Mejía | 1.6 km NE Mesa de Frías | 21.4595 | -102.8713 | 2197 | 23/09/2013 | UTADC 9940 | RRG, JLLG and JJSR |
|  | Nochistlán de Mejía | 1.4 km SE Palo Herrado | 21.3734 | -102.9493 | 2295 | 04/08/2022 | CZUAA-REP-1051 | GEQD |
| *Plestiodon obsoletus** | Mazapil | 4 km al N de Mazapil | 24.6744 | -101.5573 | 2422 | 11/06/2023 | CNAR-RF-956 | RN and ECA |
| *Aspidoscelis costatus* | Jiménez del Teúl | El Toril, 3.4 km SW Jiménez del Teúl | 23.2284 | -103.8177 | 1915 | 26/06/2012 | CZUAA-REP-902 | OVR and JJSR |
|  | Monte Escobedo | 3.7 km S Monte Escobedo | 22.2706 | -103.5498 | 2078 | 15/10/2011 | CZUAA-REP-907; CZUAA-REP-908 | MGAA, CCS and JJSR |
| *Aspidoscelis gularis* | Concepción del Oro | 1.6 km S El Salero | 24.584451 | -101.3386 | 1849 | 13/05/2015 | CNAR-RF-949 | ECA |
|  | El Salvador | 6 km NE from El Salvador Town, near tripoint Coahuila, Zacatecas, Nuevo León | 24.5313833 | -100.9518 | 2404 | 30/08/2016 | UTADC 10025 | JLLG |
|  | Mazapil | 14.4 km W El Peñasquito | 24.6478 | -101.8868 | 1665 | 07/05/2011 | CZUAA-REP-558 | JJSR |
|  | Mazapil | 11.8 km N Estación Camacho | 24.5396 | -102.3527 | 1591 | 21/06/2015 | CZUAA-REP-557 | JJSR |
|  | Pinos | 4.5 km NE from Pinos | 22.3201194 | -101.5387194 | 2637 | 20/06/2018 | UTADC 10026 | JLLG and JFMM |
|  | Villa de Cos | 2.7 km E San Andrés | 23.6875 | -101.9054 | 2120 | 13/06/2015 | CZUAA-REP-551 | JJSR |
|  | Villa García | 1.8 km NE from Villa García | 22.1718583 | -101.9504888 | 2129 | 28/06/2018 | UTADC 10027 | JLLG |
|  | Villa Hidalgo | 2.84 km NE from Villa Hidalgo | 22.3648083 | -101.6888973 | 2292 | 28/06/2018 | UTADC 10028 | JLLG |
| *Arizona elegans* | Chalchihuites | 5.1 km SE Gualterio | 23.5683 | -103.8107 | 2078 | 21/09/2012 | CZUAA-REP-401 | JJSR and OVR |
|  | Mazapil | San Juan de Cedros | 24.6593 | -101.7665 | 1768 | 22/09/2016 | CNAR-RF-942 | ECA |
|  | Mazapil | Peñasquito | 24.6186 | -101.758 | 1764 | 08/09/2015 | CNAR-RF-945 | ECA |
| *Conopsis nasus* | Genaro Codina | Cerro Gordo y Cerro Colorado, 2.1 km SE Minillas | 22.5585 | -102.4034 | 2386 | 10/10/2009 | CZUAA-REP-600 | LIAL and JJSR |
|  | Guadalupe | 12 km SE Guadalupe | 22.6785 | -102.4169 | 2224 | 02/10/2010 | CZUAA-REP-1025 | LIAL, DSCL, NYGA, SGMG and JJSR |
|  | Jerez | La Barca, Sierra de Cardos | 22.7718 | -103.1157 | 2444 | 05/09/2011 | UTADC 9941 | JLLG and JJSR |
|  | Monte Escobedo | 8.7 km W Monte Escobedo | 22.3193 | -103.6425 | 2446 | 23/09/2011 | CZUAA-REP-1023 | MGAA and JJSR |
|  | Nochistlán de Mejía | 1.2 km SE Palo Herrado | 21.3744 | -102.9518 | 2215 | 04/08/2022 | CZUAA-REP-1052 | GEQD |
|  | Pinos | 6.3 km NE Matancillas | 21.9381 | -101.6189 | 2270 | 17/09/2011 | CZUAA-REP-1026 | RVC and JJSR |
|  | Zacatecas | Zacatecas | 22.7702 | -102.5569 | 2433 | 16/10/2011 | CZUAA-REP-1024 | JLLG and JJSR |
| *Drymarchon melanurus* | Monte Escobedo | 3.7 km S Monte Escobedo | 22.2706 | -103.5498 | 2086 | 11/10/2011 | CZUAA-REP-620 | CCS, MGAA and JJSR |
|  | Moyahua de Estrada | 1.3 km NW Alameda Juárez | 21.1988 | -103.1818 | 1147 | 01/12/2010 | CZUAA-REP-615 | EDEE and JJSR |
|  | Valparaíso | San Rafael de las Tablas | 22.789 | -104.0958 | 1099 | 03/10/2022 | UTADC 9903 | JABA and JMTG |
| *Lampropeltis greeri* | Valparaíso | 2.1 km NW San José de Llanetes | 22.9217 | -103.2972 | 2214 | 21/08/2009 | CZUAA-REP-462 | JJSR |
| *Lampropeltis polyzona* | Valparaíso | 4.2 km NE San Juan Capistrano | 22.6759 | -104.0803 | 1143 | 06/08/2016 | CZUAA-REP-654 | JABA |
|  | Valparaíso | Crucero Santa Cruz, close to Nayarit | 22.4026558 | -104.340277 | 2467 | 11/08/2018 | UTADC 10038 | JLLG, MLDT and EJRP |
| *Lampropeltis splendida* | General Francisco R. Murguía | Nieves | 24.0025 | -103.0244 | 1941 | 18/09/2009 | CZUAA-REP-631 | DCSM and JJSR |
|  | Mazapil | 8 km SW El Peñasquito | 24.6345 | -101.8198 | 1678 | 27/08/2019 | CNAR-RF-951 | ECA |
| *Leptophis diplotropis** | Monte Escobedo | 8.2 km W Monte Escobedo | 22.32102 | -103.639229 | 2447 | 9/17/2011 | CZUAA-REP-443 | MGAA and JJSR |
| *Masticophis bilineatus* | Jalpa | 2.2 km W Jalpa | 21.6264 | -102.9969 | 1426 | 25/06/2016 | CZUAA-REP-921 | JJSR |
|  | Tabasco | 1.8 km NE Santiago El Chique | 22.0176 | -102.875 | 1651 | 29/09/2011 | CZUAA-REP-382 | JJSR and EMR |
|  | Villanueva | 14 km S Villanueva | 22.2317 | -102.8315 | 1983 | 21/06/2015 | CZUAA-REP-609 y CZUAA-REP-547 | JJSR, EMR and SSM |
| *Masticophis flagellum* | Guadalupe | Genaro Codina – Guadalupe road, 2.6 km S De la Riva | 22.6074 | -102.4693 | 2345 | 06/08/2010 | CZUAA-REP-1029 | JJSR |
|  | Mazapil | 3.6 km N Estación Camacho | 24.4679 | -102.365 | 1635 | 21/06/2015 | UTADC 9942 | EMR, SSN and JJSR |
|  | Villa de Cos | 8.9 km SE San Andrés | 23.6586 | -101.8532 | 2170 | 13/06/2015 | CZUAA-REP-594 | JJSR |
|  | Zacatecas | Campus UAZ Siglo XXI | 22.7708 | -102.644 | 2320 | 18/09/2009 | CZUAA-REP-397 y 601 | EEGM and JJSR |
| *Masticophis lineatus* | Momax | 6.8 km N Momax | 21.9809 | -103.2895 | 1733 | 30/06/2011 | UTADC 9943 | NYGA, LAH and JJSR |
|  | Tabasco | 3.1 km N Santiago El Chique | 22.0293 | -102.8707 | 1665 | 06/05/2011 | CZUAA-REP-1030 | JJSR |
|  | Tepechitlán | Tepechitlán | 21.6755 | -103.316 | 1733 | 18/08/2011 | UTADC 9944 | JJSR |
| *Masticophis taeniatus* | Cuauhtémoc | San Pedro Piedra Gorda | 22.453 | -102.3582 | 2065 | 09/10/2011 | UTADC 9945 | EDEE and JJSR |
|  | Genaro Codina | 2.4 km SE Genaro Codina | 22.4731 | -102.4396 | 2128 | 01/06/2012 | CZUAA-REP-608 | JJSR |
|  | Mazapil | El Peñasquito | 24.6602 | -101.7345 | 1850 | 16/08/2017 | CNAR-RF-953 | ECA |
|  | Melchor Ocampo | 2.02 km NW of Melchor Ocampo, close to the border with Coahuila | 24.8451583 | -101.6542083 | 2114 | 07/08/2018 | UTADC 10039 | JLLG |
|  | Sombrerete | 4 km S Sombrerete | 23.6013 | -103.6352 | 2267 | 21/09/2011 | CZUAA-REP-381 y CZUAA-REP-528 | JJSR and EOG |
| *Mastigodryas cliftoni* | Valparaíso | 6.6 km W San Juan Capistrano | 22.6225 | -104.1611 | 1625 | 23/09/2018 | UTADC 9899 | JABA |
| *Oxybelis microphthalmus* | Jalpa | 5.7 km N Jalpa | 21.6848 | -102.973 | 1450 | 21/06/2015 | CZUAA-REP-567 | EMR, SSM and JJSR |
|  | Juchipila | El Aguacate | 21.3684 | -103.1752 | 1469 | 19/10/2010 | CZUAA-REP-537 | JJSR |
|  | Nochistlán de Mejía | 1.6 km N El Carrizal | 21.5378 | -102.8693 | 2382 | 11/03/2011 | CZUAA-REP-442 | JJSR |
| *Pantherophis emoryi** | Mazapil | 12 km W El Peñasquito | 24.6536 | -101.8641 | 1664 | 14/10/2011 | CZUAA-REP-536 | JJSR |
| *Pituophis catenifer* | Mazapil | 1.3 km N San Rafael | 24.6033 | -102.113 | 1988 | 18/09/2010 | CZUAA-REP-595 | JJSR |
|  | Pánuco | 1.3 km E Laguna Seca | 23.0608 | -102.4811 | 2052 | 20/06/2015 | CZUAA-REP-606 | EMR, SSM and JJSR |
|  | Villa de Cos | 4.9 km E Agua Nueva | 23.7823 | -102.1126 | 2039 | 15/10/2011 | UTADC 9946 | JJSR |
| *Pituophis deppei* | Cuauhtémoc | 6.2 km NE  Genaro Codina | 22.5214 | -102.41 | 2421 | 23/08/2014 | CZUAA-REP-882 | JJSR |
|  | El Salvador | 17 km S El Salvador | 24.3772 | -100.8784 | 1814 | 02/08/2017 | UTADC 9947 | JJSR |
|  | General Pánfilo Natera | San José El Saladillo | 22.6705 | -102.051 | 2038 | 22/09/2014 | UTADC 9949 | JJSR |
|  | Guadalupe | 2.1 km SE De la Riva | 22.6127 | -102.4706 | 2338 | 29/05/2010 | CZUAA-REP-379 | JJSR |
|  | Mazapil | San Tiburcio | 24.15 | -101.5097 | 1913 | 05/07/2012 | CNAR-RF-936 | ECA |
|  | Momax | Momax | 21.9194306 | -103.31687777 | 1643 | 01/08/2015 | UTADC 10043 | JLLG |
|  | Tepechitlán | 1.48 km N La Barrenda | 21.5879 | -103.3727 | 1837 | 28/07/2011 | CZUAA-REP-1035 | JJSR |
|  | Valparaíso | 2.7 km SW Lobatos | 22.801 | -103.4192 | 2071 | 14/08/2010 | UTADC 9948 | JJSR |
|  | Villa de Cos | Primero de Mayo | 23.8043 | -101.7119 | 2026 | 13/06/2015 | CZUAA-REP-553 | JJSR |
| *Rhinocheilus lecontei* | El Salvador | 6 km NE from El Salvador Town, near tripoint Coahuila, Zacatecas, Nuevo León | 24.5631139 | -100.90128055 | 1753 | 26/08/2017 | UTADC 10045 | CEEE, JLLG and JJSR |
|  | Mazapil | Peñasquito | 24.6406 | -101.7119 | 1867 | 06/08/2015 | CNAR-RF-934 | ECA |
|  | Mazapil | Peñasquito | 24.6373 | -101.7172 | 1854 | 13/05/2017 | CNAR-RF-948 | ECA |
|  | Pánfilo Natera | El Tule | 22.6537 | -102.0253 | 2024 | 08/09/2018 | UTADC 9917 | ITAC |
| *Salvadora bairdi* | Chalchihuites | 13.1 km NE Ojo de Agua | 23.4112 | -103.7411 | 2455 | 09/11/2017 | UTADC 9918 | ITAC |
|  | Genaro Codina | Sierra Fría,8.4 km SW Genaro Codina. | 22.4216 | -102.5063 | 2341 | 27/07/2009 | CZUAA-REP-1034 | RIMV, EEGM and JJSR |
|  | Teúl de González Ortega | El Lacandón, 8.4 km SE Teúl de González Ortega | 21.4075 | -103.4076 | 1896 | 01/06/2011 | CZUAA-REP-478 | JJSR |
| *Salvadora lineata* | Melchor Ocampo | El Jaguey, 2.7 km SW Noche Buena | 24.694 | -101.6651 | 2222 | 05/08/2015 | UTADC 9920 | ITAC |
| *Senticolis triaspis* | Jalpa | 5.5 km NE Jalpa | 21.6624 | -102.9327 | 1478 | 21/06/2016 | CZUAA-REP-736, CZUAA-REP-913 | JJSR and ESM |
|  | Juchipila | 2 km S Contitlán | 21.3291 | -103.1409 | 1252 | 28/07/2011 | CZUAA-REP-441 | JJSR |
|  | Monte Escobedo | Adjuntas del Refugio | 22.6266 | -103.4131 | 1949 | 14/08/2010 | CZUAA-REP-640 | JJSR |
|  | Nochistlán de Mejía | 7.3 km SE Nochistlán de Mejía | 21.3128 | -102.8014 | 1960 | 04/08/2012 | UTADC 9950 | JJSR |
|  | Tabasco | 5 km al S Santiago El Chique | 21.967 | -102.8839 | 1608 | 29/09/2011 | CZUAA-REP-1036 | JJSR |
| *Sonora semiannulata** | Concepción del Oro | Concepción del Oro | 24.6121 | -101.4055 | 2129 | 09/10/2018 | https://www.inaturalist.org/observations/17351106 | RED |
|  | Mazapil | 6 km N General Felipe Ángeles | 24.6737 | -101.993153 | 1825 | 30/10/2016 | https://www.naturalista.mx/observations/4464755 | RO |
| *Sympholis lippiens** | Valparaíso | 12.3 km S San Juan Capistrano | 22.531 | -104.1082 | 1280 | 00/06/2016 | UTADC 9908a - 9908b | JABA |
| *Tantilla atriceps* | El Salvador | 6.1 km NW El Salvador | 24.5373 | -100.9241 | 1845 | 29/08/2017 | CZUAA-REP-843 | JLLG and JJSR |
|  | Mazapil | 14.7 km SW El Peñasquito | 24.578 | -101.8558 | 1754 | 05/09/2015 | CNAR-RF-940 | ECA |
| *Tantilla bocourti* | Jiménez del Teúl | La Cuchilla, 3.4 km SW Jiménez del Teúl | 23.2288 | -103.8186 | 1894 | 13/10/2012 | CZUAA-REP-1037 | OVR and JJSR |
| *Tantilla wilcoxi* | Genaro Codina | 6.2 km NE Genaro Codina | 22.5216 | -102.41 | 2417 | 29/08/2011 | CZUAA-REP-404 | JJSR |
|  | Monte Escobedo | 8.2 km W Monte Escobedo | 22.319386 | -103.640162 | 2440 | 23/09/2011 | CZUAA-REP-574 | MGAA and JJSR |
|  | Sombrerete | 1.4 km SE San Martin | 23.66 | -103.742 | 2620 | 06/09/2020 | UTADC 9921 | ITAC |
| *Trimorphodon tau* | Cuauhtémoc | 4.2 km NW San Pedro Piedra Gorda | 22.4851 | -102.359 | 2093 | 23/06/2011 | CZUAA-REP-1039 | JJSR |
|  | Genaro Codina | 7.2 km NE Genaro Codina | 22.5234 | -102.3993 | 2331 | 00/04/2009 | CZUAA-REP-392 | JJSR |
|  | Jerez | Cerro de las Antenas, 5.9 km SE Jerez | 22.6104 | -102.9488 | 2282 | 10/10/2014 | CZUAA-REP-628 | RARG |
|  | Nochistlán de Mejía | Puerto de la Cruces, 4.8 km W El Mirto | 21.347200 | -102.994206 | 2521 | 08/06/2006 | UTADC 9951 | DAR and JJSR |
|  | Villanueva | El Fuerte | 22.6493 | -102.7415 | 2163 | 20/06/2015 | CZUAA-REP-539 | EMR, SSM and JJSR |
| *Geophis dugesii* | Jalpa | 3 km SE La Chaveña | 21.6222 | -102.8757 | 2075 | 29/09/2011 | CZUAA-REP-417 | EMR and JJSR |
|  | Jalpa | 3.4 km SE La Chaveña | 21.6227 | -102.8675 | 2294 | 29/09/2011 | CZUAA-REP-560 | EMR and JJSR |
|  | Jalpa | 3.7 km SE La Chaveña | 21.6213 | -102.8666 | 2311 | 29/09/2011 | CZUAA-REP-466 | EMR and JJSR |
|  | Jiménez del Teúl | La presa, 7.7 km W Jiménez del Teúl | 23.2647 | -103.875 | 2139 | 27/07/2012 | CZUAA-REP-371 | OVR and JJSR |
|  | Monte Escobedo | 3.6 km S Monte Escobedo | 22.2706 | -103.5498 | 2091 | 15/10/2011 | CZUAA-REP-465 | CCS, MGAA and JJSR |
| *Heterodon kennerlyi* | Pánfilo Natera | San José el Saladillo | 22.6868 | -102.0341 | 2020 | 07/10/2020 | UTADC 9919 | ITAC |
|  | Villa Hidalgo | El Tepetate | 22.3886528 | -101.6922166 | 2271 | 19/06/2018 | UTADC 10033 | JLLG |
| *Hypsiglena jani* | El Salvador | 6 km NE from El Salvador Town, near tripoint Coahuila, Zacatecas, Nuevo León | 24.5687333 | -100.9027194 | 1753 | 26/08/2017 | UTADC 10035 | CEEE, JLLG and JJSR |
|  | Mazapil | General Felipe Ángeles | 24.6252 | -101.9803 | 1780 | 05/06/2011 | CZUAA-REP-516 | JJSR |
|  | Mazapil | 1.7 km E Coapas | 24.7744 | -102.1571 | 2044 | 20/06/2015 | CZUAA-REP-519 | EMR and JJSR |
|  | Ojocaliente | Cerro Gordo, 1.5 km NE Palmira | 22.6097 | -102.4032 | 2271 | 11/10/2009 | CZUAA-REP-1040 | LIAL and JJSR |
|  | Villa García | 2.3 km NE Villa García | 22.176 | -101.9381 | 2168 | 20/10/2014 | CZUAA-REP-584 | CEEE and JJSR |
| *Rena dugesii** | Moyahua de Estrada | 7.3 miles S (by road on Mexico Highway 54) of Santa Rosa | 21.1181 | -103.1663 | 1578 | 13/08/1984 | UTEP 10068 | RGW |
| *Storeria storerioides* | Monte Escobedo | 3.7 km SE Monte Escobedo, | 22.2706 | -103.5498 | 2087 | 15/10/2011 | CZUAA-REP-370 | CCS, MGAA and JJSR |
|  | Valparaíso | 14.8 km W Tenzompa | 22.3739 | -104.0703 | 1104 | 03/09/2018 | UTADC 9904 | JABA |
| *Thamnophis cyrtopsis* | Huanusco | 1.8 km SE San Rafael | 21.6839 | -102.7821 | 2331 | 17/06/2011 | CZUAA-REP-413 | JJSR |
|  | Monte Escobedo | 8.5 km W Monte Escobedo | 22.321 | -103.6407 | 2440 | 14/08/2010 | CZUAA-REP-1041 | MGAA |
|  | Nochistlán de Mejía | 1.7 km W El Mirto | 21.3341 | -102.968 | 2336 | 08/07/2006 | UTADC 9952 | JJSR and GEQD |
| *Thamnophis eques* | Cuauhtémoc | 7.1 km NE Genaro Codina | 22.5233 | -102.403 | 2356 | 00/08/2015 | CZUAA-REP-709 | MAL and JJSR |
|  | Genero Codina | 5.3 km W San Pedro Piedra Gorda | 22.4466 | -102.3993 | 2199 | 05/09/2010 | CZUAA-REP-1044 y 1045 | EDEE and JJSR |
|  | Jiménez del Teúl | 7.8 km W Jiménez del Teúl | 23.2647 | -103.875 | 2262 | 27/06/2012 | UTADC 9953 | OVR and JJSR |
|  | Sombrerete | Presa Joaquín Amaro, San José de Mesillas | 23.4732 | -103.5674 | 2193 | 22/09/2011 | CZUAA-REP-1043 | JJSR |
|  | Tlaltenango | 7.9 km airline SW Gral. Joaquín Amaro | 21.8954 | -103.1554 | 2615 | 14/08/2010 | SDNH_HERPPC_05452 | RACM |
| *Thamnophis melanogaster* | Chalchihuites | 2.8 km S El Pueblito | 23.3707 | -103.9102 | 2213 | 22/09/2011 | CZUAA-REP-1046 | JJSR |
|  | Genaro Codina | 2.4 km SE Genaro Codina | 22.4731 | -102.4396 | 2117 | 01/06/2012 | CZUAA-REP-1048 | OVR and JJSR |
|  | Jiménez del Teúl | 8.6 km SE Jiménez del Teúl | 23.2084 | -103.73 | 2405 | 15/07/2006 | UTADC 9954 | JJSR and JEVV |
|  | Monte Escobedo | 3.7 km S Monte Escobedo | 22.2706 | -103.5498 | 2083 | 15/10/2011 | CZUAA-REP-1047 | CCS, MGAA and JJSR |
|  | Nochistlán de Mejía | 4 km N Tlachichila | 21.5923 | -102.7846 | 2151 | 27/08/2011 | CZUAA-REP-576 | GGJ and JJSR |
|  | Pinos | Bajío del Refugio | 22.2783139 | -101.5930222 | 2357 | 23/08/2018 | UTADC 10051 | JLLG |
| *Thamnophis scaliger* | Genaro Codina | 2.3 km E Santa Inés | 22.545653 | -102.465749 | 2336 | 23/09/2011 | CZUAA-REP-415 | JJSR |
|  | Jalpa | 2.3 km E Rancho los Tres Mezquites | 21.6615 | -102.8903 | 1628 | 13/10/2011 | CZUAA-REP-883 | EDEE and JJSR |
|  | Pinos | 6.2 km N Matancillas | 21.9381 | -101.6189 | 2270 | 17/09/2011 | CZUAA-REP-607 | RVC and JJSR |
| *Indotyphlops braminus* | Guadalupe | City centre (plant retailer) | 22.7480917 | -102.5145638 | 2278 | 11/08/2018 | UTADC 10037 | JLLG |
|  | Loreto | Loreto | 22.2723 | -101.9867 | 2035 | 03/06/2018 | CZUAA-REP-710 | IVJ |
| *Crotalus pricei* | Valparaíso | Close to Ciénega de mirasoles | 22.56614 | -104.27835 | 2634 | 14/08/2020 | UAA-REP-1049 | JJSR, JJAR and JABA |
| *Crotalus scutulatus* | Loreto | El Tepetate | 22.3178 | -102.1229 | 2119 | 29/09/2017 | CZUAA-REP-785 | JJSR |
